# Chronic obstructive pulmonary disease and diabetes mellitus: a bidirectional meta-analysis of risk and comorbidity burden

**DOI:** 10.3389/fendo.2026.1912659

**Published:** 2026-08-04

**Authors:** Shuxia He, Monica Karunakaran, Rajan Rushender, Vishnu Shankar Hariharan

**Affiliations:** 1 Department of Respiratory Medicine, Shaoxing Yuecheng People’s Hospital, Shaoxing, China; 2 Department of Respiratory Medicine, Jishan Branch of Zhejiang Provincial People’s Hospital, Shaoxing, China; 3 Department of Respiratory Medicine, School of Medicine Zhejiang University, Hangzhou, China; 4 Department of Anaesthesiology, Dr MGR Educational and Research Institute, Chennai, India; 5 Department of Community Medicine, St. Peter’s Medical College Hospital and Research Institute, Hosur, India; 6 Department of Internal Medicine, Kauvery Hospital, Chennai, India

**Keywords:** chronic obstructive pulmonary disease, diabetes mellitus, meta-analysis, systematic review, type 2 diabetes

## Abstract

**Aims:**

This meta-analysis evaluated the bidirectional association and comorbidity burden between COPD and diabetes mellitus.

**Materials and methods:**

MEDLINE, Embase, Scopus, Web of Science Core Collection, and the Cochrane Library were searched from inception to May 2026. Observational studies and Mendelian randomization studies reporting comparative association estimates or prevalence data for COPD and diabetes mellitus were included. Two reviewers independently screened studies, extracted data, and assessed risk of bias using Joanna Briggs Institute tools. Random-effects meta-analyses were performed using DerSimonian–Laird inverse-variance models for association estimates and logit-transformed models for prevalence estimates.

**Results:**

Thirty-one studies met eligibility criteria. COPD was associated with higher odds of diabetes mellitus (11 studies; OR = 1.56; 95%CI, 1.37–1.79), and this association marginally persisted in adjusted analyses (OR = 1.39; 95 CI, 1.01–1.77). The pooled prevalence of diabetes mellitus among COPD patients was 17% (95%CI, 14%–21%), while type 2 diabetes mellitus prevalence was 16% (95%CI, 7%–29%). In reverse-direction analyses, diabetes mellitus was associated with increased COPD risk (12 studies; OR = 1.29; 95%CI, 1.12–1.47), but not in adjusted analyses (OR = 1.20; 95%CI, 0.82–1.57). The pooled prevalence of COPD among diabetes mellitus patients was 17% (95% CI, 14%–21%) and 19% among type 2 diabetes mellitus patients (95% CI, 11%–28%).

**Conclusion:**

This meta-analysis suggests bidirectional association between COPD and diabetes mellitus and substantial comorbidity burden, although certainty of evidence was very low because of observational evidence and substantial heterogeneity.

**Systematic review registration:**

https://www.crd.york.ac.uk/PROSPERO/view/CRD420261413229, identifier CRD420261413229.

## Introduction

Chronic obstructive pulmonary disease (COPD) is a common, preventable, and treatable chronic respiratory disease characterized by persistent airflow limitation, chronic respiratory symptoms, and substantial heterogeneity in clinical presentation (1). Recent global estimates continue to demonstrate a considerable COPD burden, particularly among older adults and individuals exposed to tobacco smoke, biomass fuel, occupational particles, and ambient air pollution (2). Although COPD is defined by abnormalities of the airways and lung parenchyma, its clinical expression extends beyond the respiratory system. Contemporary descriptions of COPD emphasize its systemic consequences, including skeletal-muscle dysfunction, reduced physical activity, chronic inflammation, and frequent coexistence with cardiometabolic comorbidities (3). This broader disease phenotype is especially relevant because many patients with COPD are older, have cumulative exposure to shared environmental and behavioural risk factors, and require long-term pharmacological treatment that may influence metabolic health (4).

Diabetes mellitus is also increasing worldwide and represents one of the most important chronic non-communicable diseases affecting adult populations. A recent pooled global analysis estimated that 828 million adults were living with diabetes in 2022, reflecting a marked rise over the past three decades (5). The coexistence of COPD and diabetes mellitus is therefore not merely a chance finding in ageing populations; it may reflect overlapping risk profiles and interrelated biological pathways. Smoking, air pollution, obesity, socioeconomic disadvantage, poor diet, and physical inactivity are relevant to both disorders. In addition, COPD itself may contribute to metabolic vulnerability through chronic systemic inflammation, hypoxaemia, oxidative stress, neurohormonal activation, reduced skeletal-muscle oxidative capacity, and loss of lean muscle mass (6, 7). These mechanisms can promote insulin resistance and impaired glucose homeostasis, particularly among patients with more advanced respiratory symptoms, limited exercise tolerance, or repeated inflammatory insults (7).

The relationship between COPD and diabetes may also operate in the reverse direction. Hyperglycaemia and diabetes can affect the lung through advanced glycation end-products, endothelial dysfunction, pulmonary microangiopathy, impaired collagen turnover, autonomic neuropathy, and altered immune responses (6, 7). These changes may influence lung structure, gas exchange, host defence, and vulnerability to respiratory infections. Oxidative stress is a central mechanism in COPD pathobiology and may also amplify insulin resistance and pancreatic beta-cell dysfunction, creating a feed-forward inflammatory–metabolic axis (8). Treatment-related factors further complicate this association. Systemic glucocorticoids, frequently used during COPD exacerbations, are strongly associated with new-onset hyperglycaemia and diabetes, while inhaled corticosteroids may have dose- and duration-dependent glycaemic effects in selected patients (9, 10). Thus, diabetes among individuals with COPD may arise from shared exposures, disease-mediated metabolic effects, and treatment-related mechanisms rather than from a single causal pathway (11).

Epidemiological evidence increasingly supports an association between COPD, impaired lung function, and diabetes mellitus, although the magnitude of this relationship has varied across settings. A meta-analysis of cohort studies reported that COPD and reduced lung function were associated with a higher risk of subsequent type 2 diabetes (12). Conversely, a large prospective UK Biobank study found that diabetes, prediabetes, and longer diabetes duration were associated with increased risk of COPD (13). Genetic epidemiology has also contributed to this field, with a recent Mendelian randomization study reporting evidence consistent with COPD as a causal risk factor for type 2 diabetes (14). However, reported estimates may be influenced by differences in COPD definition, diabetes ascertainment, smoking adjustment, obesity and cardiometabolic confounding, corticosteroid exposure, disease severity, and study design (11).

Clarifying the relationship between COPD and diabetes mellitus is clinically important because both conditions are common, chronic, and frequently managed through separate care pathways. More precise quantification of diabetes burden among individuals with COPD, and of the comparative association between COPD and diabetes across populations, may support integrated cardiometabolic assessment, glycaemic monitoring, and risk-factor modification in COPD care. The present meta-analysis was therefore undertaken to synthesize available observational evidence on the bidirectional association between COPD and diabetes mellitus, focusing on the burden of diabetes among individuals with COPD and the strength of association between COPD and diabetes in comparative populations.

## Methods

This review was conducted and reported in accordance with the Preferred Reporting Items for Systematic Reviews and Meta-Analyses 2020 statement and checklist (15).

### Eligibility criteria

Studies were considered eligible using a population–exposure–comparator–outcome–study design framework. Observational studies evaluating the association between chronic obstructive pulmonary disease and diabetes mellitus were included. Eligible study designs comprised cohort studies, case-control studies, cross-sectional studies, and baseline observational analyses of interventional studies if relevant data were reported separately. Studies were eligible if they reported diabetes mellitus among individuals with COPD, compared diabetes mellitus between COPD and non-COPD populations, or individuals with diabetes mellitus compared COPD between diabetes mellitus and non-diabetes mellitus populations, or reported COPD among individuals with diabetes mellitus or reported diabetes mellitus among individuals with COPD in a manner that allowed extraction of prevalence or comparative association estimates.

The population of interest included adults aged 18 years or older from community, primary-care, hospital-based, registry-based, or administrative database settings. Studies including mixed adult and paediatric populations were included only when adult data were reported separately or when the study population was predominantly adult. COPD was accepted as defined by the original investigators, including spirometry-based definitions, physician diagnosis, International Classification of Diseases diagnostic codes, medical record diagnosis, validated registry definitions, or self-reported physician-diagnosed COPD. Diabetes mellitus was accepted when defined using physician diagnosis, medical records, diagnostic codes, antidiabetic medication use, laboratory criteria such as fasting plasma glucose or glycated haemoglobin, registry data, or self-report. Studies reporting type 1 or type 2 or mixed type diabetes mellitus were included. Studies reporting unspecified diabetes mellitus were also included and analysed as diabetes mellitus unless the report clearly referred to gestational diabetes, steroid-induced transient hyperglycaemia only, or another non-comparable condition.

Studies were excluded if they were reviews, editorials, commentaries, case reports, case series without extractable prevalence or association data, animal studies, *in vitro* studies, genetic-only studies without clinical COPD or diabetes ascertainment, or studies in which COPD was combined with asthma or other respiratory diseases without separable COPD data. Studies focused exclusively on acute hyperglycaemia during COPD exacerbation were excluded unless chronic diabetes mellitus was reported separately. Conference abstracts were included only if they provided sufficient extractable data; otherwise, they were listed as excluded at full-text review with the reason “insufficient data”.

### Information sources and search strategy

Systematic search was conducted from database inception to May 2026 in MEDLINE, Embase, Scopus, Web of Science Core Collection, and Cochrane library. Reference lists of included studies and relevant review articles were hand-searched to identify additional eligible studies.

The search strategy combined controlled vocabulary terms and free-text keywords for COPD and diabetes mellitus. Search terms included variants of “chronic obstructive pulmonary disease,” “COPD,” “chronic obstructive lung disease,” “emphysema,” “chronic bronchitis,” “diabetes mellitus,” “type 2 diabetes,” “T2DM,” “hyperglycaemia,” and “glycaemic control.” Database-specific subject headings, including MeSH and Emtree terms, were adapted for each database. The search strategy was designed to maximize sensitivity and was not restricted by study design filters unless required by specific database interface. The complete search strategy for each database is provided in **Supplementary File 1**.

### Study selection process

**Figure 1:**
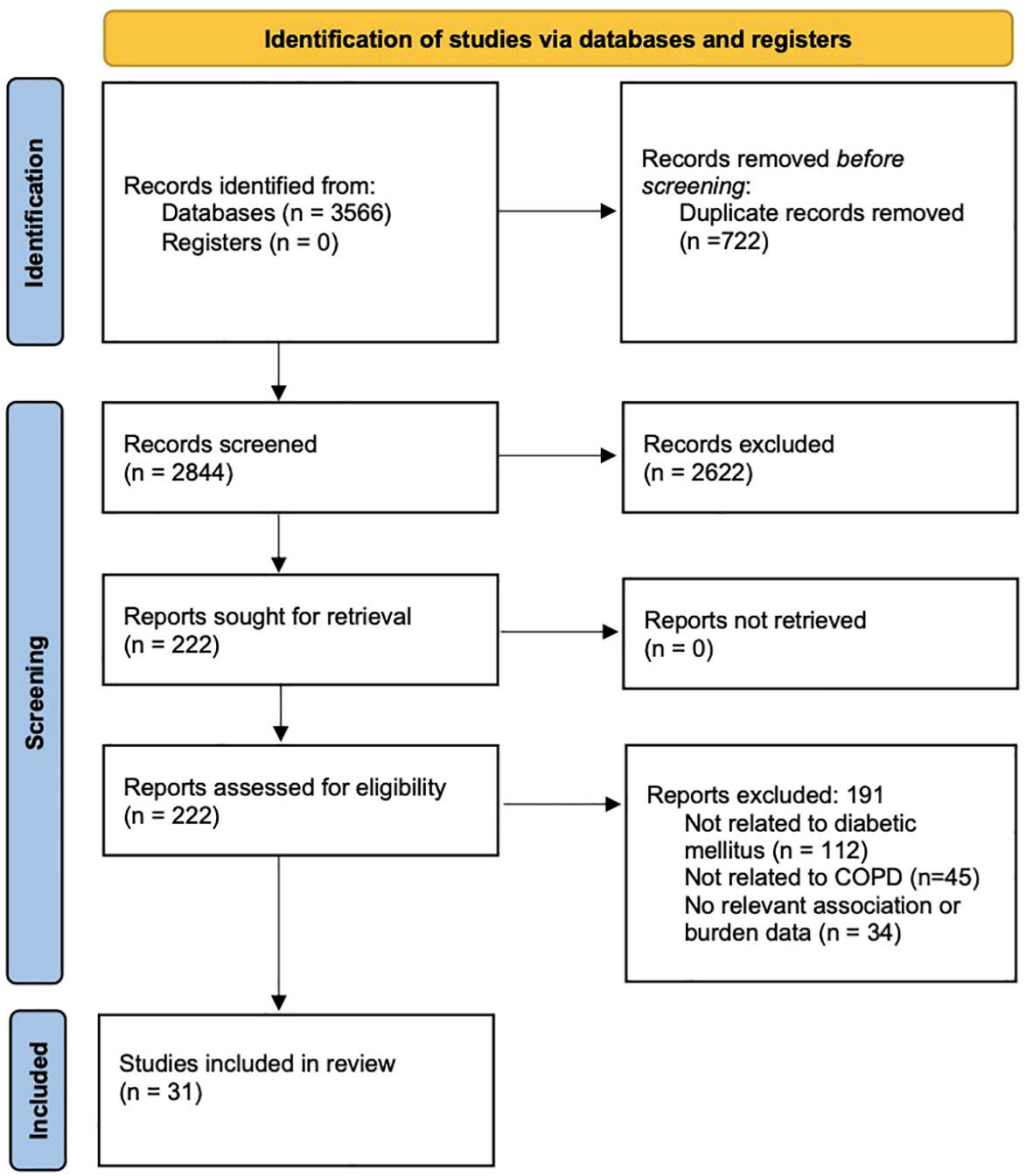
PRISMA 2020 flow diagram of study selection. Flow diagram showing identification, screening, eligibility assessment, and inclusion of studies evaluating the association between chronic obstructive pulmonary disease and diabetes mellitus.

All records identified through database searching were imported into Rayyan for de-duplication and then for screening. Duplicate records were removed electronically and manually verified. Two reviewers independently screened titles and abstracts against the eligibility criteria. Full texts of potentially eligible articles were then retrieved and assessed independently by the same two reviewers. Disagreements at either stage were resolved through discussion; unresolved disagreements were adjudicated by third reviewer. Reasons for exclusion at the full-text stage were recorded and are reported in the PRISMA 2020 flow diagram. No automation tool was used to make final inclusion or exclusion decisions (**Figure 1**).

### Data collection process

Data were extracted independently by two reviewers using standardized, pilot-tested data extraction form. The form was piloted on sample of eligible studies and refined before full extraction. Extracted data were compared between reviewers, and discrepancies were resolved through consensus or third-reviewer adjudication. Where necessary, study authors were contacted for missing or unclear data. When authors did not respond, available published data were used. If multiple reports described overlapping populations, the report with the largest sample size, longest follow-up, most complete adjustment, or most relevant data for given synthesis was prioritized. When overlapping reports contributed different non-duplicative data, each was used only for the relevant analysis to avoid double counting.

### Data items

The following data were extracted from each included study: first author, year of publication, country, geographical region, study design, setting, recruitment period, data source, sample size, population characteristics, age, sex distribution, smoking status where available, body mass index where available, COPD definition, COPD severity where reported, diabetes definition, diabetes type, method of diabetes ascertainment, comparator definition, number of participants with COPD, number of participants with diabetes mellitus, crude event counts, prevalence estimates, adjusted and unadjusted effect estimates, confidence intervals, variables included in adjusted models, follow-up duration for cohort studies, funding source, and author-reported conflicts of interest. When studies reported several adjusted models, the most fully adjusted estimate was extracted for the primary synthesis.

### Risk of bias assessment

Two reviewers independently assessed the methodological quality and risk of bias of included studies. The Joanna Briggs Institute critical appraisal tools appropriate to each study design were used for cohort, case-control, cross-sectional, and prevalence studies, with attention to sampling methods, representativeness, exposure ascertainment, outcome ascertainment, confounding, completeness of data, and appropriateness of statistical analysis.

Specifically, we applied JBI Checklist for Cohort Studies (11 domains), JBI Checklist for Case-Control Studies (10 domains), JBI Checklist for Analytical Cross-Sectional Studies (8 domains), and the JBI Checklist for Studies Reporting Prevalence Data (9 domains) to their respective study designs. Methodological quality was quantified based on the proportion of ‘Yes’ responses scored across the design-specific domains. Studies achieving a ‘Yes’ score ≥70% were classified as having a low risk of bias; scores between 50% and 69% were classified as moderate risk of bias; and scores <50% were categorized as high risk of bias. Disagreements during the appraisal process were thoroughly adjudicated via consensus between the two independent reviewers (16).

### Effect measures

For prevalence analyses, the effect measure was the proportion of individuals with diabetes mellitus among participants with COPD and the proportion of individuals with COPD among participants with diabetes mellitus, expressed as prevalence with 95% confidence intervals (CIs). Where studies reported COPD among individuals with diabetes mellitus, these estimates were analysed separately and were not combined with diabetes-prevalence estimates among COPD populations. Comparative analyses was constructed by extracting raw dichotomous data specifically, the number of events and total participants from the included studies. From these 2x2 data matrices, we calculated unadjusted Odds Ratios (ORs) with 95% CIs. Effect estimates were pooled on the logarithmic scale and back-transformed for presentation.

### Data synthesis and statistical analysis

A narrative synthesis was first performed to describe study characteristics, COPD and diabetes definitions, study populations, and patterns of reported associations. Meta-analysis was undertaken when at least two studies were sufficiently similar in terms of population, exposure definition, outcome definition, study design, and effect measure. Separate syntheses were performed for diabetes prevalence among COPD patients, COPD prevalence among diabetes patients and for comparative association estimates between COPD and diabetes mellitus bidirectionally.

Crucially, while different observational and genetic designs evaluate distinct aspects of disease association with varying susceptibilities to bias they were pooled in separate, stratified meta-analyses rather than singular omni-analysis. Pooling observational designs (cohort, case-control, cross-sectional) alongside Mendelian randomization within separate syntheses serves to triangulate epidemiological evidence. Triangulation across distinct methodologies with orthogonal biases (e.g., residual confounding in observational studies versus pleiotropy in MR) provides a more comprehensive assessment of robustness. To maintain methodological rigor, study design was explicitly treated as a primary stratifying variable in prespecified subgroup analyses to systematically evaluate how structural design differences influenced the pooled estimates.

Because substantial clinical and methodological heterogeneity was anticipated, random-effects models were used for meta-analysis. For prevalence estimates, pooled proportions were calculated using logit-transformed random-effects model, with results back-transformed to prevalence estimates for interpretation. Comparative effect estimates were pooled using inverse-variance random-effects meta-analysis on the log scale. Between-study variance was estimated using DerSimonian–Laird method. Statistical analyses were performed using STATA version 18.

Forest plots were generated to display study-specific and pooled estimates. Statistical heterogeneity was assessed using Cochran’s Q test, the I² statistic, and tau-squared. I² values were interpreted in the context of the number of studies, magnitude and direction of effects, and clinical diversity rather than by rigid thresholds alone. Prediction intervals were calculated when appropriate to describe the expected range of effects in future comparable settings. Prespecified subgroup analyses were conducted where data permitted according to study design, geographical region, setting and diabetes type. Subgroup analyses were interpreted as exploratory. Meta-regression was considered when at least ten studies were available for a covariate. Leave-one out sensitivity analyses were performed to evaluate robustness of the findings (17).

### Assessment of reporting bias and small-study effects

For meta-analyses including at least ten studies, funnel plots were generated to assess small-study effects, and Egger’s regression test was used for funnel-plot asymmetry where appropriate. Funnel-plot asymmetry was interpreted cautiously because asymmetry may arise from heterogeneity, differences in study size, population risk, study quality, or selective reporting rather than publication bias alone. Formal tests for funnel-plot asymmetry were not performed when fewer than ten studies were available.

### Certainty of evidence

The certainty of evidence for the main pooled estimates was assessed using the Grading of Recommendations Assessment, Development and Evaluation approach. Certainty was evaluated across the domains of risk of bias, inconsistency, indirectness, imprecision, and publication bias. Because the included evidence was observational, comparative evidence was initially rated as low certainty and was downgraded or upgraded according to GRADE criteria where appropriate. Certainty ratings were summarized as high, moderate, low, or very low. For prevalence estimates, certainty was judged using the same core domains, with particular attention to representativeness of the sampled population, validity of COPD and diabetes ascertainment, heterogeneity, and precision of pooled estimates.

### Ethical considerations

Ethical approval was not required because this study used aggregate data from previously published studies and did not involve collection of individual participant data.

## Results

### Study selection process

A total of 3,566 records were identified through database searches, of which 722 duplicates were removed before screening. After title and abstract screening, 2,622 records were excluded, and 222 full-text reports were assessed for eligibility. Of these, 191 reports were excluded because they were not related to diabetes mellitus, not related to COPD, or did not provide relevant association or burden data. Finally, 31 studies met the eligibility criteria and were included (12, 13, 18–46).

### Characteristics of the included studies

The characteristics of the included studies are summarized in **Table 1**. Of the 31 included studies, 10 were prospective cohort studies, 9 were retrospective cohort studies, 9 were cross-sectional studies, 2 were Mendelian randomization studies, and one case-control study. The studies were conducted across diverse geographical settings, most commonly in the USA (n = 7), Taiwan (n = 4), Spain (n = 3), and South Korea (n = 3), with additional studies from China, UK, Japan, Nordic countries, Sweden, India, Denmark, Italy, Finland, Bulgaria, Greece, and multi-country datasets.

**Table 1:** Characteristics of the included studies (N=31).

Study (year)	Study design	Country	Characteristics of participants	Diagnostic criteria	Adjustment variables	Disease severity	Sample size	Mean age	Male/female	ROB
Chen (2024) (18)	Mendelian Randomisation	Japan	Type 2 diabetes cases and COPD cases from an East Asian population for a Mendelian randomization analysis.	GWAS summary data (Japanese Biobank Project); physician-confirmed diagnosis	Age, age, gender, height, principal components, and smoking habits	NR	210,865	NR	NR	Low
Chiu (2017) (19)	Retrospective Cohort	Taiwan	Diabetic patients aged over 30 years who were free of COPD at baseline.	DM: ADA criteria (ICD-9-CM 250); COPD: ≥ 1 inpatient or ≥ 3 outpatient claims (ICD-9-CM 490–496)	Age, gender, smoking, alcohol, duration of diabetes, hypoglycemic drug type, hypertension meds, glucocorticoids, obesity, baseline glucose/HbA1c, and various comorbidities	NR	27,257	60.4	13,000 M / 14,257 F	Moderate
Correa-Gutiérrez (2023) (20)	Prospective Cohort	Spain	Patients older than 40 years of age hospitalized due to an exacerbation of COPD.	COPD: Personal history, symptoms, and spirometry; DM: Physician diagnosis	Descriptive analysis used for direct comparison; no multivariate risk models reported for primary association	Mean FEV1pp 46.7% (Severe)	50	70.5	30 M / 20 F	Low
Ehrlich (2010) (21)	Retrospective Cohort	USA	Health plan members aged ≥ 18 years with or without a diagnosis of diabetes.	DM: Diabetes registry (A1C ≥ 6.7%, meds, or dx); COPD: ICD-9 codes (hospitalisation or cause of death)	Age, sex, race/ethnicity, smoking, BMI, education, alcohol consumption, and number of outpatient visits	Severe (hospitalized or death)	1,811,228	NR	53.1% M (DM)	High
Feary (2009) (22)	Cross-sectional	UK	General primary care patients aged >35 years identified from a large UK database.	Physician-diagnosed COPD and DM identified via primary care general population database	Age, smoking status, and Townsend deprivation score	GOLD stages: Mild to Very Severe	1,204,110	NR	618,090 M / 586,020 F	High
Figueira (2021) (23)	Prospective Cohort	Spain	Active or former smokers aged over 40 years with spirometry-confirmed COPD (FEV1/FVC < 0.7; FEV1 ≤ 65%).	COPD: Spirometry-confirmed (FEV1/FVC < 0.7; FEV1 ≤ 65%); DM: T2DM clinical history	Age, FEV1 (% predicted), exacerbator phenotype, pneumococcal vaccination status, and cardiovascular disease	Moderate to Severe (FEV1 ≤ 65%)	121	72.0	105 M / 16 F	Low
Golpe (2024) (24)	Retrospective Cohort	Spain	Consecutive COPD outpatients diagnosed according to GOLD criteria.	COPD: Outpatients diagnosed according to GOLD criteria; DM: HbA1c and clinical history	Cox models adjusted for multiple clinical parameters and DM control groups	Mean FEV1% 52.3% [Source data]	1,124	68.6	895 M / 229 F	Low
Gudmundsson (2006) (25)	Prospective Cohort	Nordic	Patients hospitalized for acute exacerbations of COPD in Nordic countries.	Hospitalized for acute COPD exacerbation; cause of death by ICD-10	Health status, medications, and various co-morbidities	GOLD I-IV	416	72.1	216 M / 200 F	Low
Gunasekaran (2021) (26)	Retrospective Cohort	USA	Hospitalized adults (aged ≥ 18 years) who had a primary diagnosis of COPD.	Adults ≥ 18 years with primary diagnosis of COPD (ICD-9-CM codes) in NIS database	Clinical and demographic characteristics and comorbidities	Hospitalized (Acute Exacerbation)	7,498,577	68.9	3.3M M / 4.2M F	Moderate
Ho (2019) (27)	Retrospective Cohort	Taiwan	Adults aged ≥ 40 years with spirometry-confirmed COPD (FEV1/FVC < 0.7).	COPD: FEV1/FVC < 0.7; DM: ICD codes plus antidiabetics OR glucose/HbA1c criteria	Age, sex, BMI, hypertension, cerebrovascular disease, heart failure, coronary artery disease, malignancy, CKD, FEV1, and prior hospitalizations	GOLD stage 1–4	4,231	72.0	3,525 M / 706 F	High
Joo (2012) (28)	Cross-sectional	South Korea	Subjects aged ≥ 40 yr who met ATS/ERS recommendation criteria for spirometry.	COPD: Aged ≥ 40 yr, FEV1/FVC < 0.7; DM: Meds OR fasting glucose ≥ 126 mg/dL	Age, sex, BMI, waist circumference, smoking status, CRP, and HOMA-IR	Mild to Moderate	2,177	64.6	238 M / 116 F	High
Kamour (2015) (29)	Cross-sectional	USA	Adult residents 18 years and older reporting their COPD and comorbidity status.	Self-reported COPD and diabetes (KyBRFS population survey)	Age, level of education, income, and smoking status	NR	9,585	55.2	3,302 M / 6,283 F	Moderate
Kim (2017) (30)	Cross-sectional	South Korea	Participants aged ≥ 40 years from a nationwide representative population.	COPD: FEV1/FVC < 0.70; DM: Fasting glucose ≥ 126, HbA1c ≥ 6.5%, meds, or physician dx	Age, sex, smoking status, exercise, dyslipidemia medication, diabetes duration, and log-transformed triglycerides	Obstructive pattern present	8,784	54.3	4,409 M / 4,375 F	Moderate
Krishnan (2024) (31)	Retrospective Cohort	USA	Patients aged 40–89 years with COPD matched 1:1 to a non-COPD cohort.	COPD and DM: ≥ 2 diagnoses ≥ 30 days apart in Optum CDM database	Age-matching (decades), weight status, and presence of underweight status	NR	316,212	71.1	135,666 M / 180,542 F	Low
Lee (2013) (32)	Retrospective Cohort	Taiwan	COPD patients matched 1:1 with age- and gender-matched control subjects.	COPD: ICD-9 491, 492, 496; DM: ICD-9 250 plus ≥ 1 claim within 12 months	Gender, age, residential area, socioeconomic status, steroid use, hypertriglycemia, hypertension, coronary artery disease, and cerebrovascular disease	Standard COPD diagnosis	16,088	67.9	11,668 M / 4,420 F	Low
Lee (2020) (33)	Cross-sectional	South Korea	Subjects aged 40 or older who participated in the Korea National Health and Nutrition Examination Survey.	COPD: FEV1/FVC < 0.7; DM: HbA1c ≥ 6.5% or fasting glucose ≥ 126 mg/dL	Age, sex, BMI, waist circumference, smoking status, C-reactive protein, and HOMA-IR	Obstructive Pulmonary Disease	2,830	54.6	1,446 M / 1,384 F	High
Lin (2017) (34)	Retrospective Cohort	Taiwan	Adults aged ≥ 40 years newly diagnosed with COPD exacerbations or COPD without exacerbations.	COPD: ICD-9-CM 491, 492, 496; DM: ICD-9-CM 250	Sociodemographics, comorbidities (e.g., hypertension, mental disorders, heart disease), and medications	Stable vs. Exacerbation	32,699	59.0	16,499 M / 16,200 F	Moderate
Lin (2021) (35)	Prospective Cohort	China	Patients hospitalized with a confirmed diagnosis of acute exacerbation of COPD.	COPD: Clinical dx + FEV1/FVC < 0.70; T2DM: Meds OR fasting ≥ 7.0 OR random ≥ 11.1 mmol/L	Age, PaO2, smoking status, gender, and respiratory failure	AECOPD (Hospitalized)	196	72.1	129 M / 67 F	Low
Lindberg (2011) (36)	Cross-sectional	Sweden	Individuals with COPD according to GOLD spirometric criteria (FEV1/FVC < 0.70) and matched controls.	COPD: FEV1/FVC < 0.7; DM: Physician diagnosis or antidiabetic treatment	Gender, BMI, smoking habits, and age	GOLD Stage I to IV	1,986	64.5	1,079 M / 907 F	High
Mahishale (2015) (37)	Cross-sectional	India	Subjects aged more than 40 years diagnosed with COPD as per GOLD criteria.	COPD: Post-bronchodilator FEV1/FVC < 70%; DM: WHO criteria (FBS > 126 or PPBS > 200)	Multivariate regression using GOLD category as a predictor for DM	Mild, moderate, severe, and very severe	2,432	NR	1,648 M / 784 F	High
Mao (2021) (38)	Prospective Cohort	China	Hospitalized inpatients aged 18 years or older with a confirmed diagnosis of AECOPD.	AECOPD: Symptoms + FEV1/FVC < 0.7; DM: WHO 1999 criteria or prior diagnosis	Age group, sex, smoking, hospital level, ICU admission, disease duration, insurance, CAT score, steroids, and various comorbidities	GOLD stage 1–4	5,334	69.0	4,201 M / 1,133 F	Low
Meteran (2015) (39)	Case-Control	Denmark	Twins aged 50–71 years from the nation-wide Danish Twin Registry.	COPD: Hospitalized patients (FEV1/FVC < 0.70); DM: WHO 2008 criteria	Regression analysis for Metabolic Syndrome components (e.g., fasting glucose)	Mild, moderate, severe, and very severe	13,649	59.0	6,415 M / 7,234 F	Moderate
Rana (2004) (40)	Prospective Cohort	USA	Women aged 30–55 years who were free of preexisting type 2 diabetes at baseline.	COPD: ICD-8 and ICD-10 codes; T2DM: ICD-8 and ICD-10 codes	Sex, age group, smoking, and BMI	Based on ICD codes	97,618	58.0	0 M / 97,618 F	Low
Rogliani (2014) (41)	Cross-sectional	Italy	Outpatients with COPD attending a respiratory diseases clinic in Italy.	COPD: Physician-diagnosed/validated by questionnaire; DM: National Diabetes Data Group criteria	Age, BMI, sedentary status, smoking status, alcohol intake, and dietary score	Standard COPD diagnosis	493	72.1	336 M / 157 F	Moderate
Song (2010) (42)	Prospective Cohort	USA	Female health professionals aged ≥ 45 years who were free of diabetes at baseline.	COPD: Clinical history + spirometry (FEV1/FVC < 0.70); DM: Confirmed clinical history	BMI and GOLD stage	GOLD Mild, Moderate, and Severe	38,570	57.8	0 M / 38,570 F	Low
Su (2023) (13)	Prospective Cohort	UK	Participants aged 40–69 years who were without COPD at baseline.	COPD: Self-reported (emphysema, chronic bronchitis, bronchiectasis); DM: Expert Committee criteria	Age, randomized treatment, BMI, smoking, physical activity, alcohol, hormones, and dietary factors	Self-reported	452,680	56.5	204,611 M / 248,069 F	Low
Wang (2024) (14)	Mendelian Randomisation	Multi country	European ancestry COPD cases and East Asian type 2 diabetes cases from GWAS summary data.	COPD: ICD-10 (J40–J44); DM: Self-report + HbA1c ≥ 6.5%	Age, sex, ethnicity, deprivation index, BMI, smoking, alcohol, exercise, family history, meds, glucose, and asthma	ICD-10 confirmed	691,351	NR	NR	Low
Annavarapu (2018) (46)	Retrospective Cohort	USA	Medicare Advantage and commercially insured patients with COPD aged 55–89 years.	COPD: Prebronchodilator spirometry (FEV1 < 80% pred; FEV1/FVC < 0.7); DM: Multiple glucose/meds criteria	Age, gender, height, principal components, smoking habits, and BMI	FEV1 < 80% and FEV1/FVC < 0.7	45,722	71.4	18,151 M / 27,571 F	High
Koskela (2014) (45)	Prospective Cohort	Finland	Adult patients admitted to a pulmonology ward for acute exacerbation of asthma or COPD.	COPD: ≥ 2 claims with ICD-9-CM diagnosis code; DM: Type 2 DM comorbidity	Stepwise logistic regression (demographic, geographic, treatment, and utilization variables)	Severe Exacerbations (Hospitalized) vs. Non-severe	43 (COPD)	NR	24 M / 19 F (COPD)	Low
Mekov (2015) (44)	Cross-sectional	Bulgaria	Hospitalized COPD patients with post-bronchodilator spirometric obstruction (FEV1/FVC < 0.70).	Pulmonologist-confirmed COPD; DM: Doctor's dx or screening (HbA1c ≥ 6.5%)	Age, BMI, Karnofsky score, presence of COPD, oxygen saturation, and urea	Mild to Moderate Exacerbation	152	65.0	108 M / 44 F	High
Papaioannou (2021) (43)	Prospective Cohort	Greece	Consecutive patients admitted for an acute exacerbation of COPD aged over 40 years.	COPD: Post-bronchodilator FEV1/FVC < 0.7; DM: Type 2 DM medical history	NR	Acute Exacerbation (AECOPD)	156	73.0	116 M / 40 F	Low

COPD, Chronic Obstructive Pulmonary Disease; DM, Diabetes Mellitus; T2DM, Type 2 Diabetes Mellitus; AECOPD, Acute Exacerbation of Chronic Obstructive Pulmonary Disease; FEV1, Forced Expiratory Volume in One Second; FVC, Forced Vital Capacity; GOLD, Global Initiative for Chronic Obstructive Lung Disease; ATS, American Thoracic Society; ERS, European Respiratory Society; GWAS, Genome-Wide Association Study; NR, Not Reported; M, Male; F, Female; Int., International; UK, United Kingdom; USA, United States of America; COPDGene, Genetic Epidemiology of COPD Study; MRI, Magnetic Resonance Imaging; RLN, Recurrent Laryngeal Nerve; SAH, Subarachnoid Hemorrhage; ICU, Intensive Care Unit; LMIC, Low- and top-Income Countries; HIC, High-Income Countries; NPC, Nasopharyngeal Carcinoma; NP, Nasopharyngeal; NPLH, Nasopharyngeal Lymphoid Hyperplasia; FU, Follow-up; EUA, Examination Under Anaesthesia.

The reported sample sizes varied substantially, ranging from 43 participants in the smallest COPD cohort to 7,498,577 participants in the largest retrospective database study. The median sample size was 8,784 participants. Among studies reporting mean age, the mean age ranged from 54.3 to 73.0 years, indicating that most included populations consisted of middle-aged to older adults. Sex distribution was reported in most studies, although two studies did not report sex-specific data and one study reported only the percentage of males. Several studies included predominantly male COPD populations, whereas two prospective cohort studies included women only.

With respect to study populations, included studies comprised community-based samples, primary-care or administrative database cohorts, hospital-based COPD or acute exacerbation cohorts, outpatient COPD cohorts, and genetic summary-data analyses. COPD was commonly defined using physician diagnosis, administrative codes, spirometry-confirmed airflow obstruction, GOLD criteria, or hospitalization for COPD or acute exacerbation of COPD. Based on risk-of-bias assessment, 15 studies were judged to have low risk of bias, 7 had moderate risk of bias, and 9 had high risk of bias.

### Risk of diabetes mellitus amongst COPD patients

**Figure 2:**
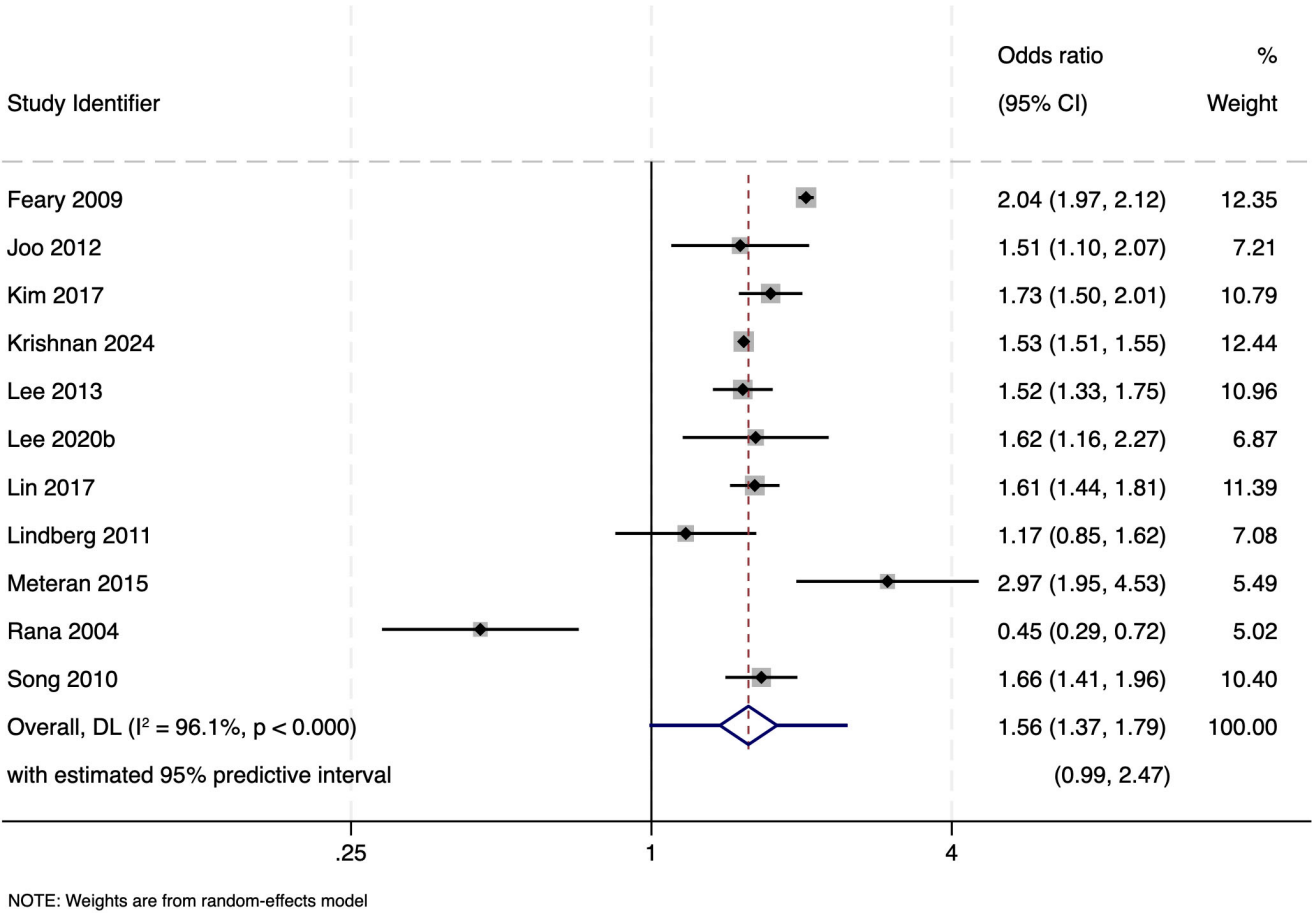
Forest plot of the risk of diabetes mellitus among patients with COPD. Random-effects meta-analysis of odds ratios comparing diabetes mellitus risk among patients with COPD versus non-COPD populations.

**Figure 3:**
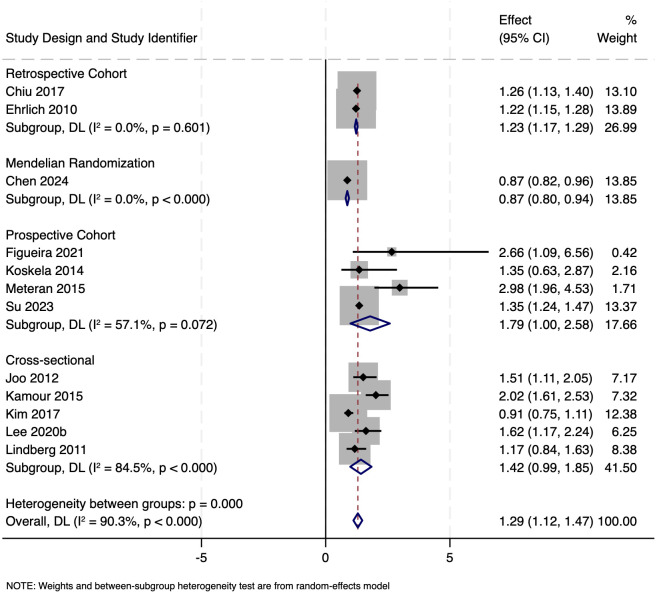
Subgroup analysis of COPD risk among diabetes mellitus patients by study design. Random-effects subgroup meta-analysis stratified by retrospective cohort, Mendelian randomization, prospective cohort, and cross-sectional study design.

As shown in **Figures 2**, **3** studies evaluating 1,729,366 participants were included in the meta-analysis of diabetes mellitus risk among patients with COPD. Using a DerSimonian–Laird random-effects model, COPD was associated with significantly higher odds of diabetes mellitus, with pooled OR of 1.56 (95% CI: 1.37–1.79; p < 0.001).

Most included studies demonstrated an increased odds of diabetes among COPD patients, although individual study estimates varied considerably. Substantial heterogeneity was observed across studies (I² = 96.1%; p < 0.001), and the estimated 95% prediction interval ranged from 0.99 to 2.47, indicating that the magnitude of association may vary across future comparable populations.

#### Subgroup analysis

##### Study design

**Figure 4:**
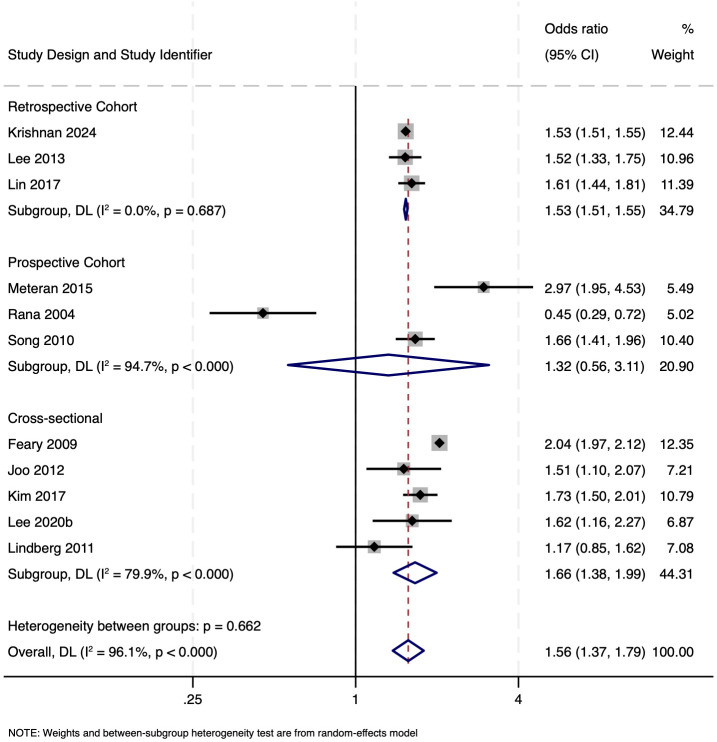
Subgroup analysis of diabetes mellitus risk among COPD patients by study design. Random-effects subgroup meta-analysis stratified by retrospective cohort, prospective cohort, and cross-sectional study design.

In subgroup analysis by study design (**Figure 4**), association between COPD and diabetes mellitus remained significant in retrospective cohort studies (OR: 1.53; 95% CI: 1.51–1.55; I² = 0.0%) and cross-sectional studies (OR: 1.66; 95% CI: 1.38–1.99; I² = 79.9%). Among prospective cohort studies, the pooled estimate showed a positive but non-significant association with wide confidence intervals (OR: 1.32; 95% CI: 0.56–3.11; I² = 94.7%). There was no statistically significant difference between subgroups by study design (p for subgroup difference = 0.662).

##### Geographical region

**Figure 5:**
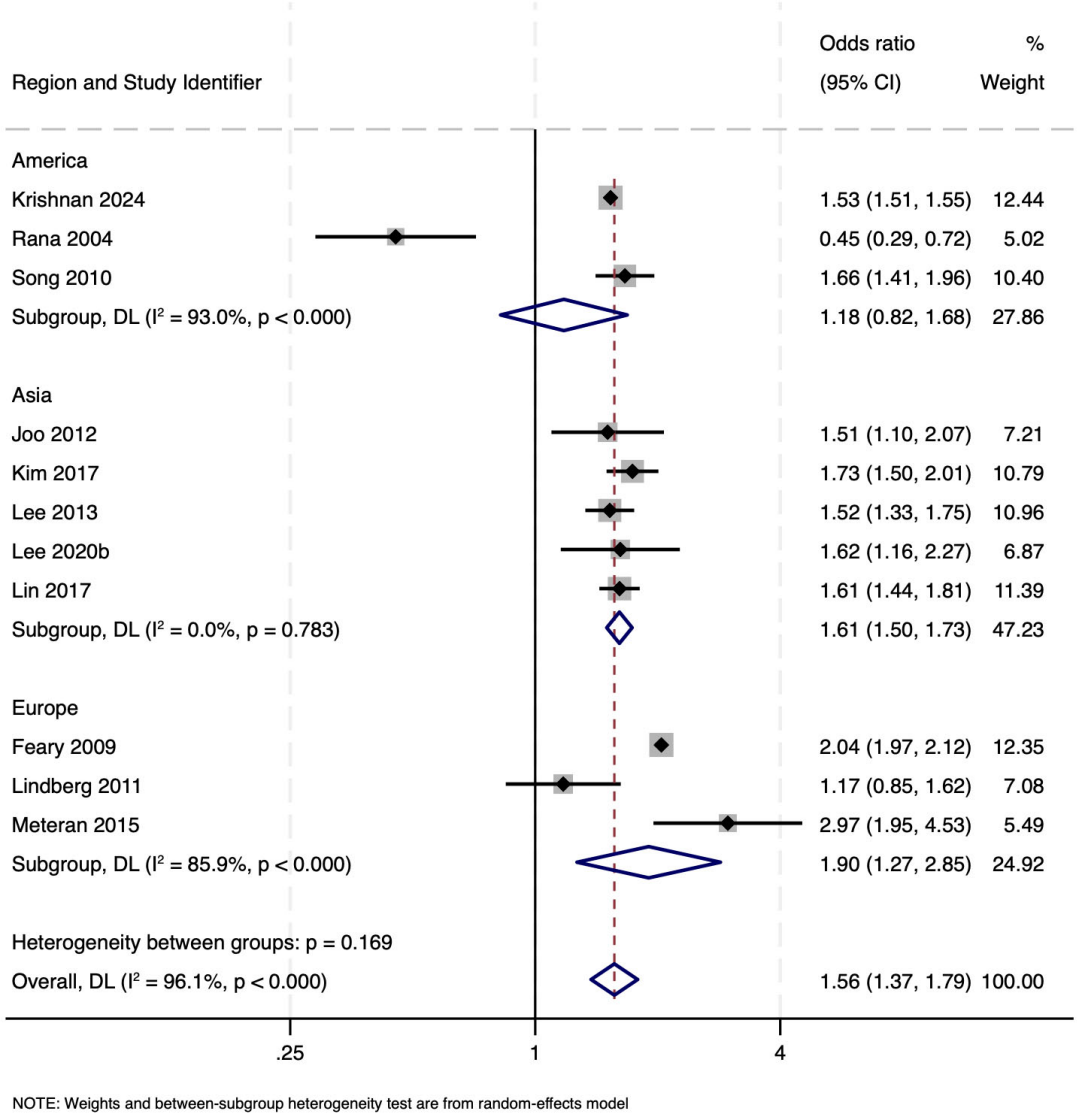
Subgroup analysis of diabetes mellitus risk among COPD patients by geographical region. Random-effects subgroup meta-analysis stratified by studies conducted in America, Asia, and Europe.

In subgroup analysis by geographical region (**Figure 5**), association between COPD and diabetes mellitus was significant in studies from Asia (OR: 1.61; 95% CI: 1.50–1.73; I² = 0.0%) and Europe (OR: 1.90; 95% CI: 1.27–2.85; I² = 85.9%). The pooled estimate for studies from America showed positive but non-significant association (OR: 1.18; 95% CI: 0.82–1.68; I² = 93.0%). However, the test for subgroup differences was not statistically significant (p = 0.169), indicating no clear evidence that the association differed by geographical region.

##### Type of diabetes

**Figure 6:**
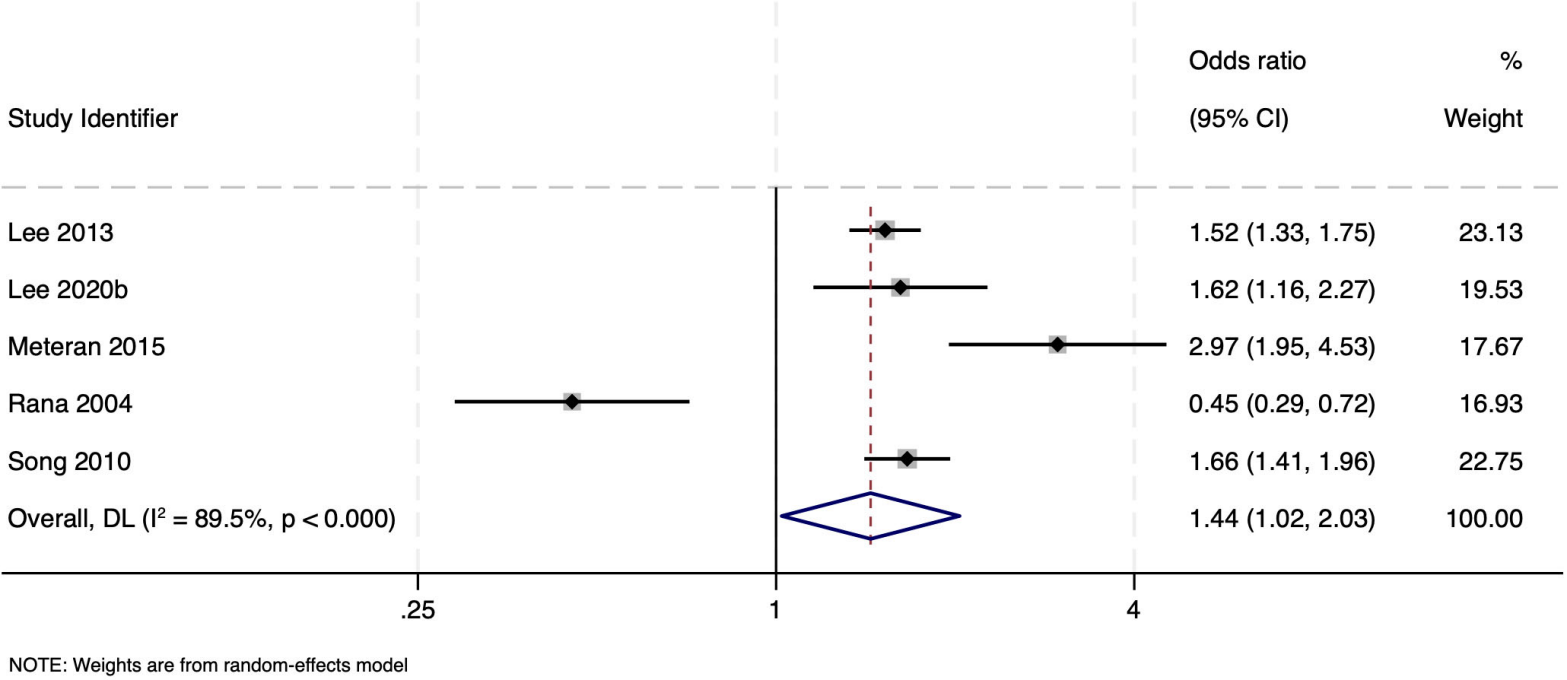
Forest plot of the risk of type 2 diabetes mellitus among patients with COPD. Random-effects meta-analysis restricted to studies specifically reporting type 2 diabetes mellitus among COPD patients.

In the analysis restricted to studies specifically reporting type 2 diabetes mellitus among COPD patients, 5 studies were included (**Figure 6**). Using a DerSimonian–Laird random-effects model, COPD was associated with significantly higher odds of type 2 diabetes mellitus, with pooled OR of 1.44 (95% CI: 1.02–2.03). Considerable heterogeneity was observed across studies (I² = 89.5%; p < 0.001), suggesting substantial variability in the magnitude and direction of the association.

All the studies assessing this relationship was done as community based studies. Hence, subgroup analysis by study setting was not possible.

#### Adjusted analysis

**Figure 7:**
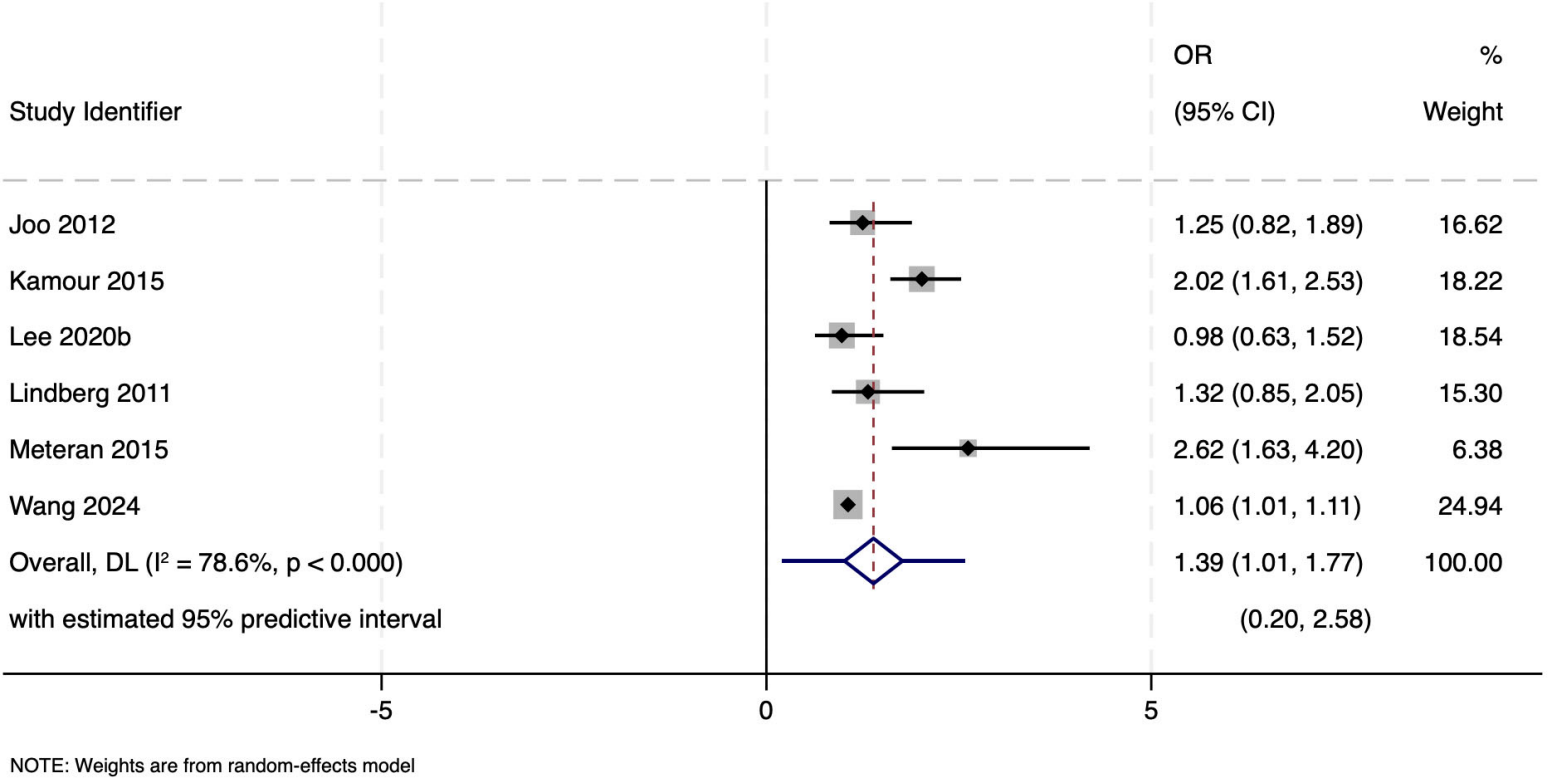
Forest plot of adjusted effect estimates for diabetes mellitus risk among COPD patients. Random-effects meta-analysis restricted to adjusted association estimates reported by the included studies.

In the analysis restricted to adjusted OR, six studies were included to evaluate the risk of diabetes mellitus among patients with COPD (**Figure 7**). The pooled adjusted OR showed that COPD was marginally associated with increased risk of diabetes mellitus (OR: 1.39; 95% CI: 1.01–1.77).

Most studies reported positive association after adjustment for confounders, with individual adjusted estimates ranging from 0.98 to 2.62. Moderate-to-substantial heterogeneity was observed across studies (I² = 78.6%; p < 0.001), and the estimated 95% prediction interval was 0.20 to 2.58, suggesting that the strength of association may vary across different populations and study settings.

#### Additional analysis

Univariable meta-regression was done using the potential covariates such as study design, geographical region, type of diabetes and risk of bias findings. None of the variables were able to explain the substantial heterogeneity obtained in the pooled estimates. Leave-one out sensitivity analysis (**Supplementary Figure 1**) showed that there was no significant variation in terms of direction and strength of association. Egger’s regression test did not indicate significant small-study effects or funnel-plot asymmetry (bias coefficient = 0.59; 95% CI: −3.79 to 4.97; p = 0.768) (**Supplementary Figure 2**).

### Burden of diabetes mellitus amongst COPD patients

**Figure 8:**
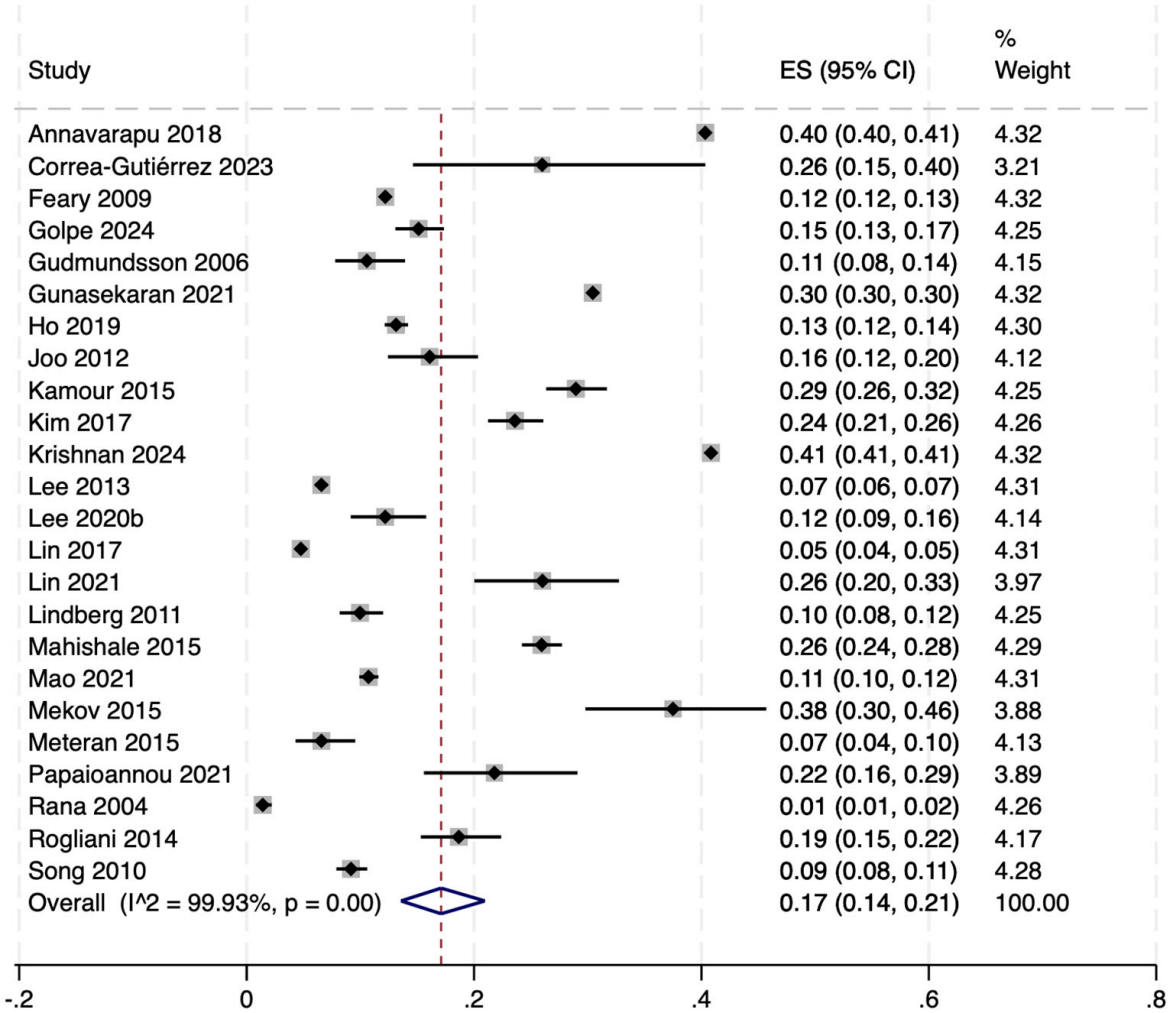
Forest plot of the pooled prevalence of diabetes mellitus among patients with COPD. Random-effects meta-analysis of prevalence estimates reporting the burden of diabetes mellitus in COPD populations.

For the analysis of the burden of diabetes mellitus among patients with COPD, 24 studies contributed prevalence data. The pooled prevalence of diabetes mellitus among COPD patients was 17% (95% CI: 14%–21%) (**Figure 8**). Considerable heterogeneity was observed across studies (I² = 99.93%; p < 0.001).

**Figure 9:**
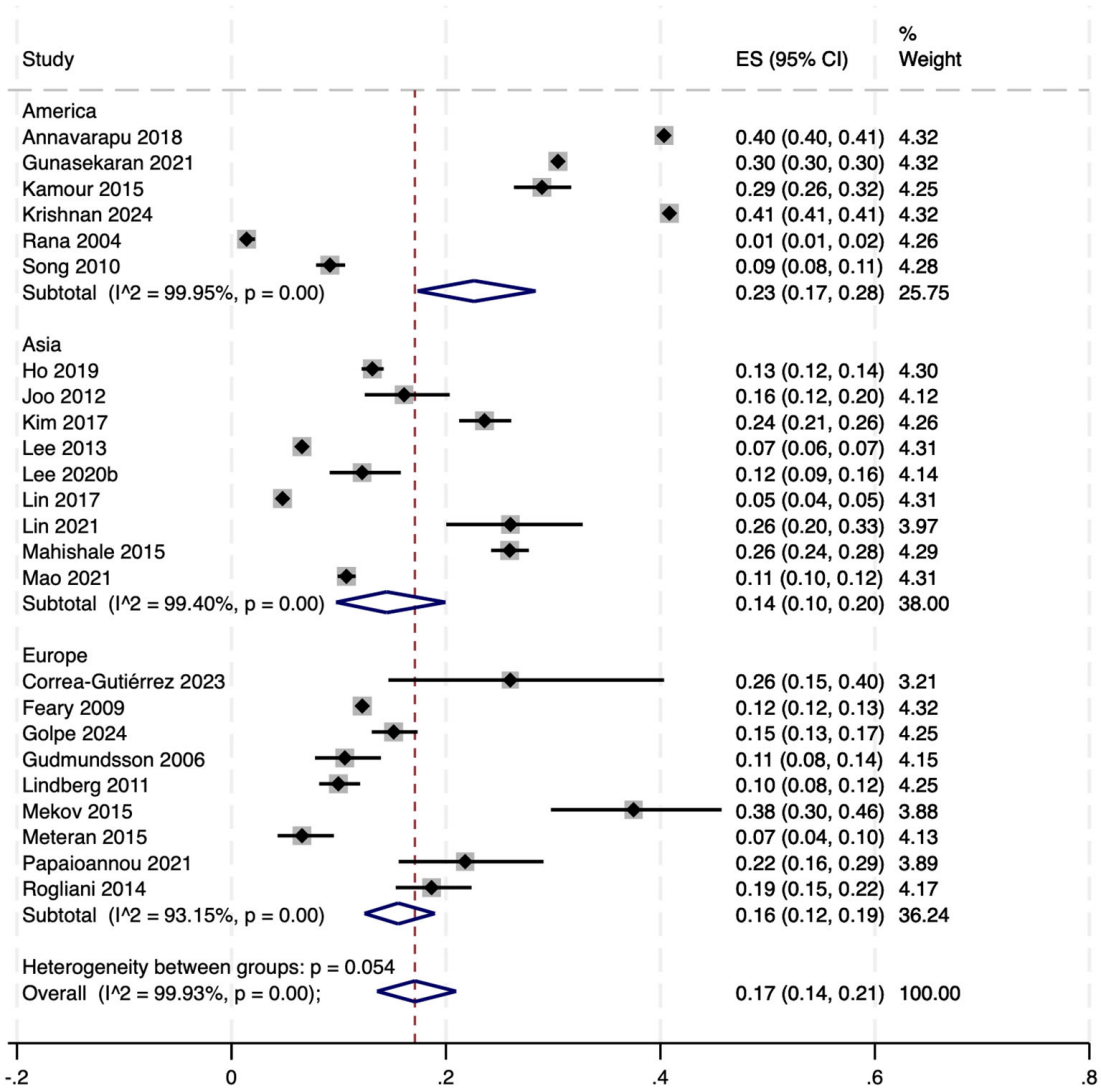
Subgroup analysis of diabetes mellitus prevalence among COPD patients by geographical region. Random-effects subgroup meta-analysis of diabetes mellitus burden among COPD patients stratified by America, Asia, and Europe.

In subgroup analysis by geographical region, the pooled prevalence of diabetes mellitus among COPD patients was highest in America (23%; 95% CI: 17%–28%; 6 studies; I² = 99.95%), followed by Europe (16%; 95% CI: 12%–19%; 9 studies; I² = 93.15%) and Asia (14%; 95% CI: 10%–20%; 9 studies; I² = 99.40%) (**Figure 9**). The test for between-region subgroup differences approached but did not reach statistical significance (p = 0.054).

**Figure 10:**
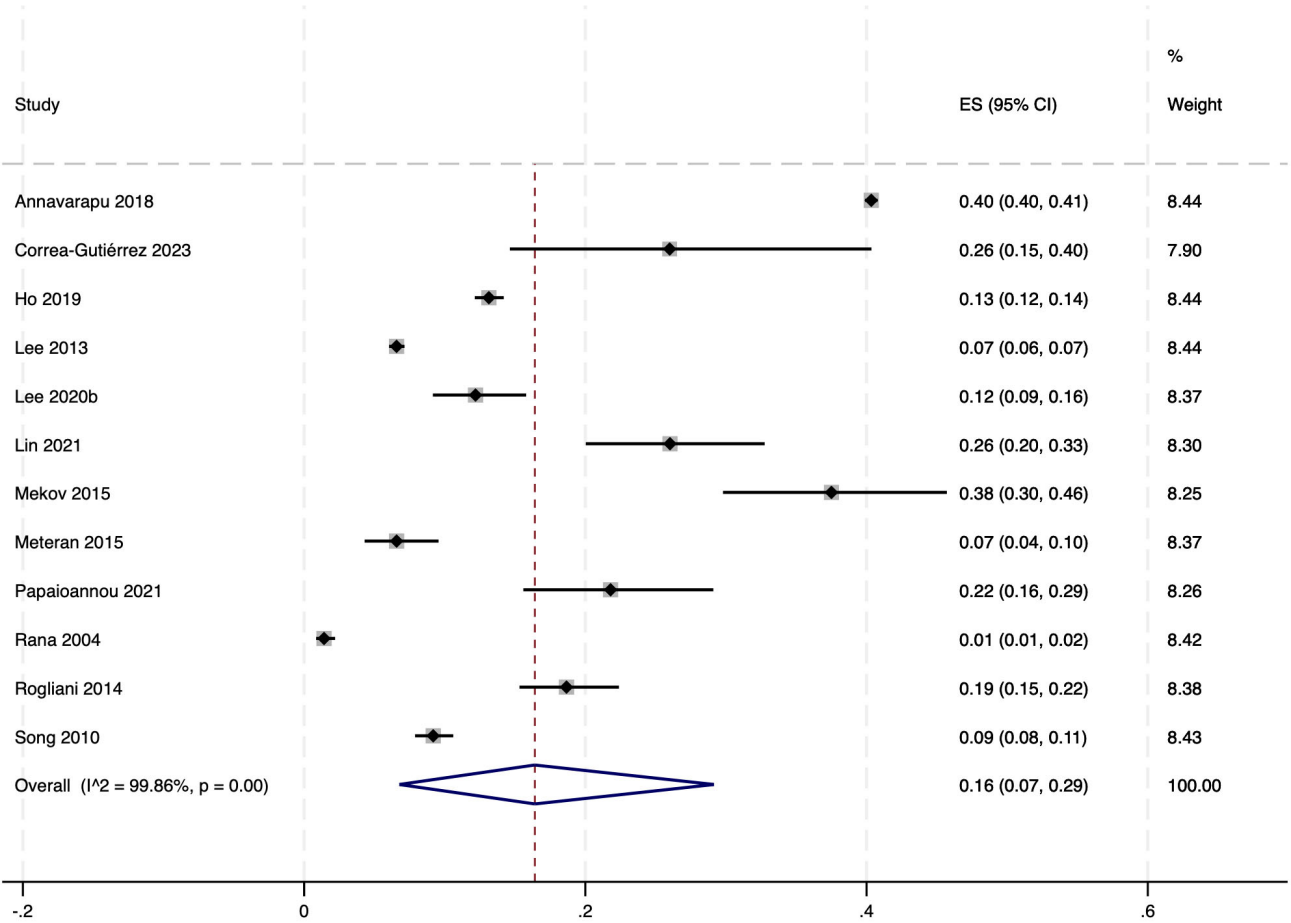
Forest plot of the pooled prevalence of type 2 diabetes mellitus among patients with COPD. Random-effects prevalence meta-analysis restricted to studies reporting type 2 diabetes mellitus in COPD populations.

In the analysis restricted to studies reporting type 2 diabetes mellitus among patients with COPD, 12 studies contributed prevalence data. The pooled prevalence of type 2 diabetes mellitus among COPD patients was 16% (95% CI: 7%–29%) (**Figure 10**).

### Risk of COPD amongst diabetes mellitus patients

**Figure 11:**
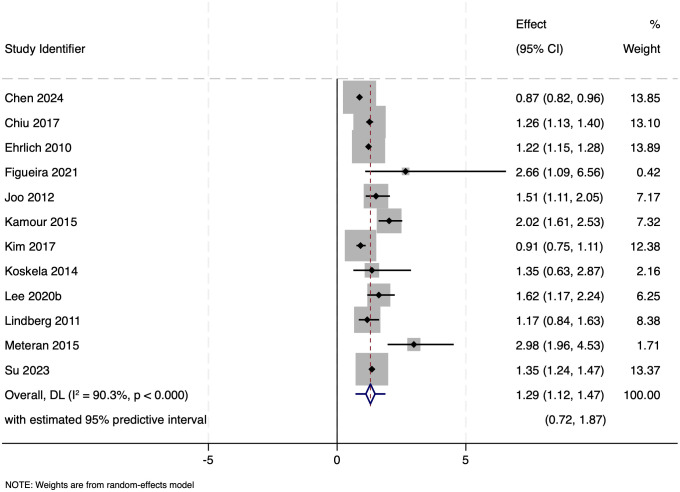
Forest plot of the risk of COPD among patients with diabetes mellitus. Random-effects meta-analysis evaluating the reverse-direction association between diabetes mellitus and subsequent or prevalent COPD.

In the reverse-direction analysis evaluating the risk of COPD among patients with diabetes mellitus, 12 studies were included. Analysis showed that diabetes mellitus was associated with significantly higher risk of COPD, with pooled OR of 1.29 (95% CI: 1.12–1.47) (**Figure 11**). Substantial heterogeneity was observed across studies (I² = 90.3%; p < 0.001), and the estimated 95% prediction interval was 0.72 to 1.87, indicating that the direction and magnitude of the association may vary across future comparable populations.

#### Subgroup analysis

##### Study design

In subgroup analysis by study design, the association between diabetes mellitus and COPD risk varied significantly across subgroups (p for subgroup difference < 0.001) (**Figure 3**). Retrospective cohort studies showed significant positive association with low heterogeneity (OR: 1.23; 95% CI: 1.17–1.29; I² = 0.0%), while prospective cohort studies showed a stronger, borderline association (OR: 1.79; 95% CI: 1.00–2.58; I² = 57.1%). Cross-sectional studies showed a positive but non-significant pooled estimate (OR: 1.42; 95% CI: 0.99–1.85; I² = 84.5%). Mendelian randomization subgroup, represented by one study, showed an inverse association (OR: 0.87; 95% CI: 0.80–0.94).

##### Geographical region

**Figure 12:**
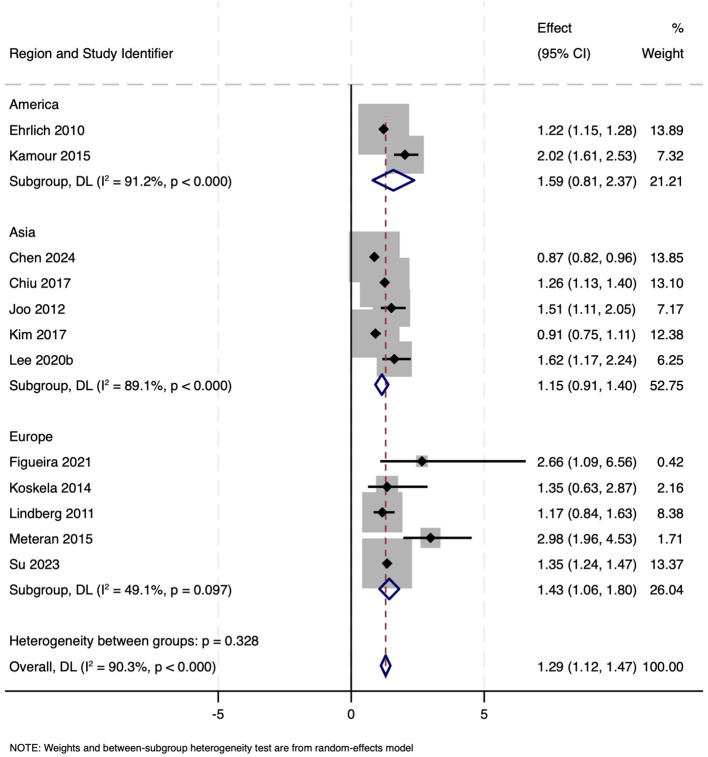
Subgroup analysis of COPD risk among diabetes mellitus patients by geographical region. Random-effects subgroup meta-analysis stratified by studies conducted in America, Asia, and Europe.

In subgroup analysis by geographical region, the pooled association between diabetes mellitus and COPD risk was significant in European studies (OR: 1.43; 95% CI: 1.06–1.80; I² = 49.1%). The pooled estimates for American studies (OR: 1.59; 95% CI: 0.81–2.37; I² = 91.2%) and Asian studies (OR: 1.15; 95% CI: 0.91–1.40; I² = 89.1%) were positive but not statistically significant. There was no statistically significant difference between geographical subgroups (p for subgroup difference = 0.328) (**Figure 12**).

##### Study setting

**Figure 13:**
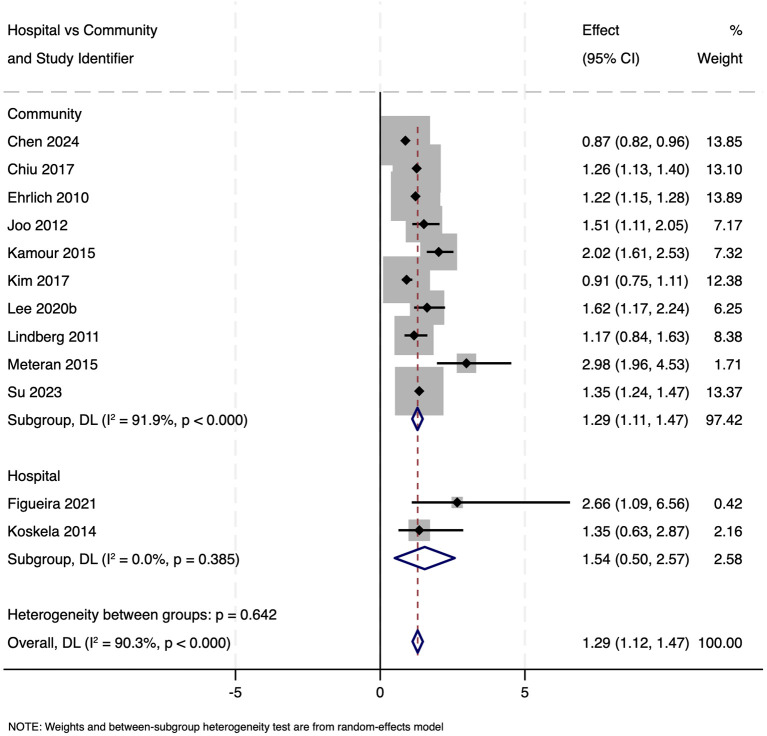
Subgroup analysis of COPD risk among diabetes mellitus patients by study setting. Random-effects subgroup meta-analysis comparing community-based and hospital-based study populations.

In subgroup analysis by study setting, the pooled association between diabetes mellitus and COPD risk was significant in community-based studies (OR: 1.29; 95% CI: 1.11–1.47; I² = 91.9%). In contrast, pooled estimate for hospital-based studies was higher in magnitude but not statistically significant, with wide confidence intervals (OR: 1.54; 95% CI: 0.50–2.57; I² = 0.0%). There was no statistically significant difference between community- and hospital-based subgroups (p for subgroup difference = 0.642) (**Figure 13**).

##### Type 2 diabetes mellitus

**Figure 14:**
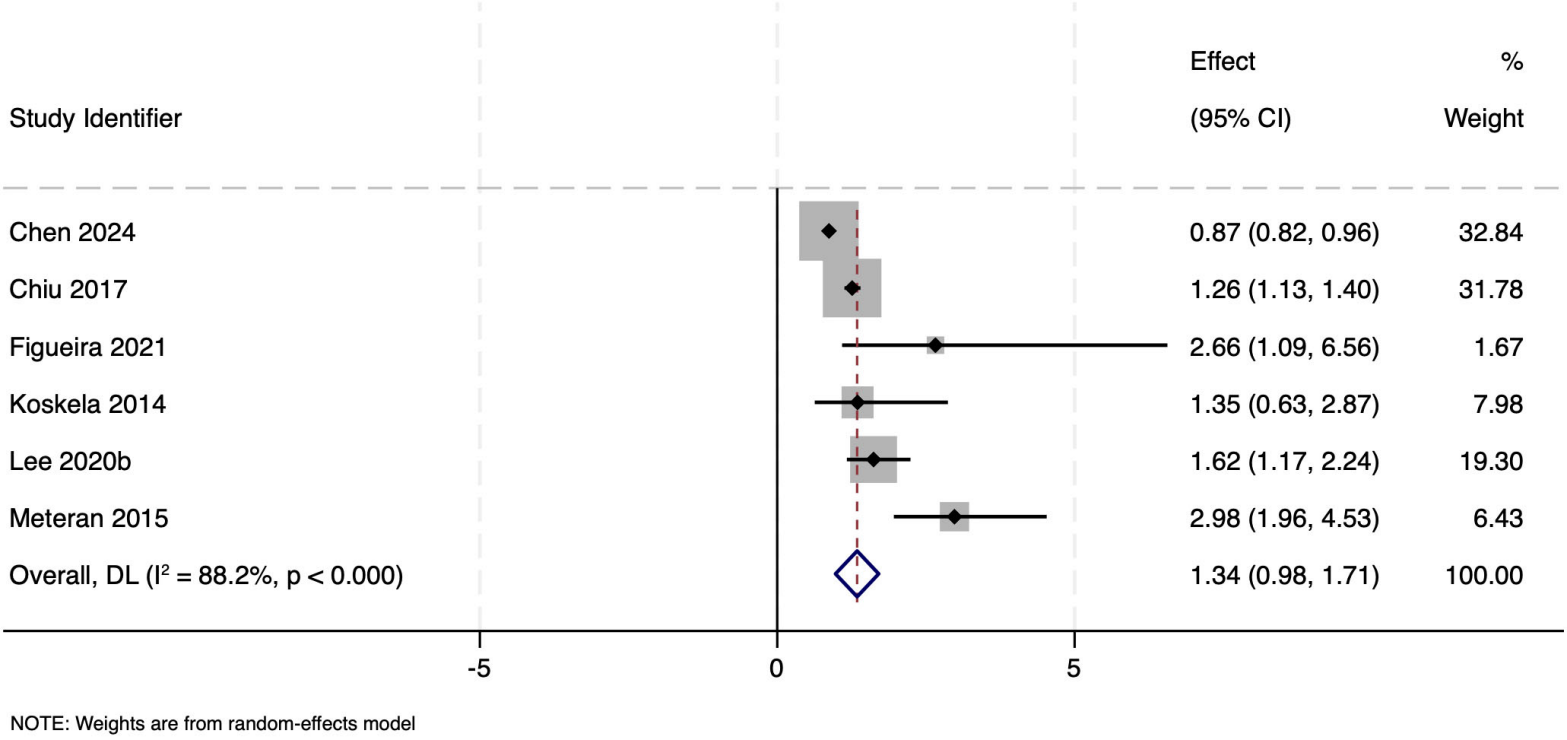
Forest plot of the risk of COPD among patients with type 2 diabetes mellitus. Random-effects meta-analysis restricted to studies specifically evaluating type 2 diabetes mellitus as the exposure.

In the analysis restricted to studies specifically evaluating type 2 diabetes mellitus, 6 studies were included to assess the risk of COPD (**Figure 14**). The pooled random-effects estimate suggested a positive association, but this did not reach statistical significance (OR: 1.34; 95% CI: 0.98–1.71). Substantial between-study heterogeneity was found (I² = 88.2%; p < 0.001).

#### Adjusted analysis

**Figure 15:**
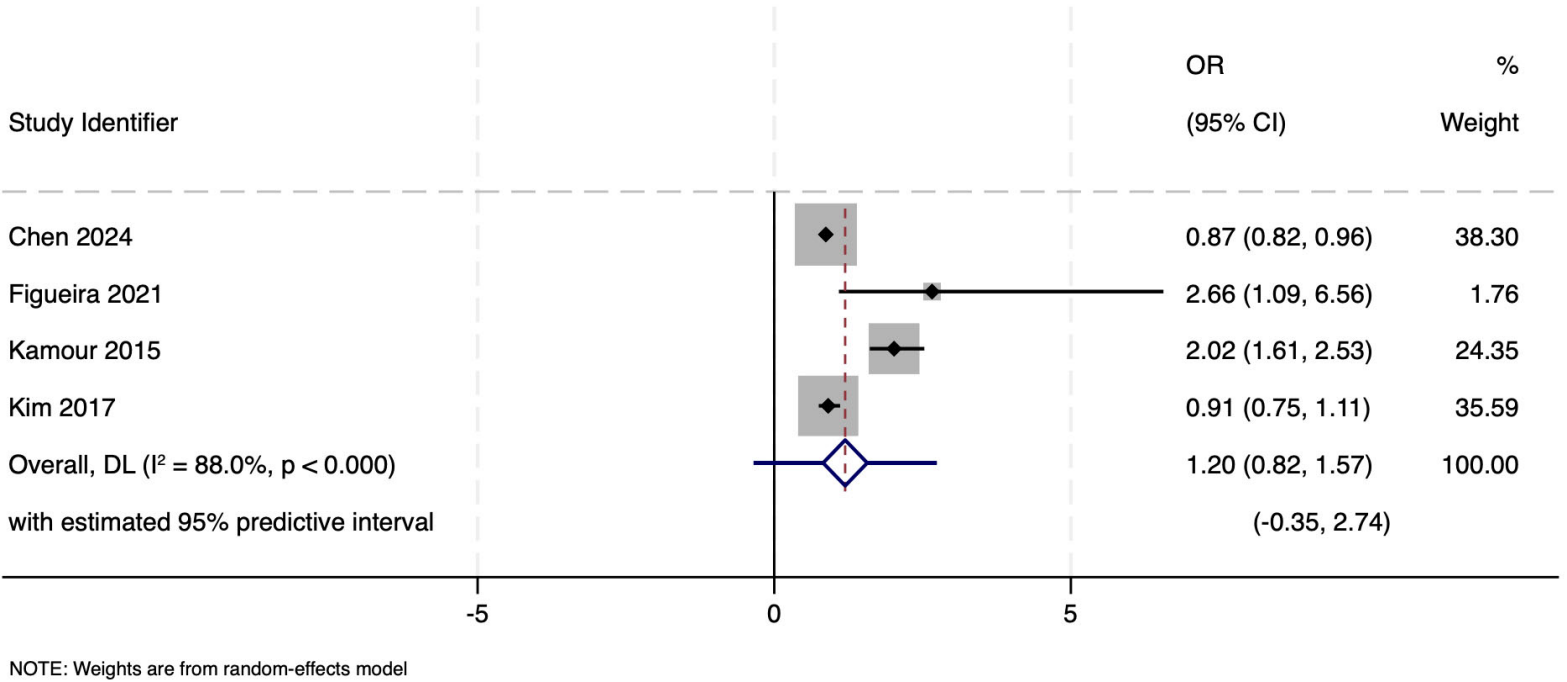
Forest plot of adjusted effect estimates for COPD risk among diabetes mellitus patients. Random-effects meta-analysis restricted to adjusted association estimates reported by the included studies.

In the analysis restricted to adjusted OR, four studies were included to evaluate the risk of COPD among patients with diabetes mellitus. The pooled OR showed that diabetes mellitus was not significantly associated with increased risk of COPD (OR: 1.20; 95% CI: 0.82–1.57) (**Figure 15**). Substantial heterogeneity was observed across studies (I² = 88.0%; p < 0.001).

#### Additional analysis

Univariable meta-regression was done using the potential covariates such as study design, geographical region, study setting, type of diabetes and risk of bias findings. None of the variables were able to explain the substantial heterogeneity obtained in the pooled estimates. Leave-one out sensitivity analysis (**Supplementary Figure 3**) showed that there was no significant variation in terms of direction and strength of association. Publication bias for the overall analysis was assessed using Egger’s regression test across 12 studies (**Supplementary Figure 4**). The test did not show statistically significant evidence of small-study effects or funnel-plot asymmetry (bias coefficient = 1.96; 95% CI: −0.82 to 4.74; p = 0.147).

### Burden of COPD amongst diabetes mellitus patients

Overall, 10 studies contributed data on the burden of COPD among patients with diabetes mellitus. The pooled random-effects estimate showed overall COPD prevalence of 17% among diabetes mellitus patients (95% CI: 14%–21%) (**Supplementary Figure 5**). Substantial heterogeneity was observed across studies (I^2^ = 99.29%; p<0.001).

In subgroup analysis by geographical region, pooled prevalence of COPD among diabetes mellitus patients was 18% in Asian studies (95% CI: 9%–28%; I^2^ = 98.59%) and 16% in European studies (95% CI: 12%–20%; I^2^ = 98.94%) (**Supplementary Figure 6**). There was no statistically significant difference between regional subgroups (p=0.776). In subgroup analysis by study setting, the pooled prevalence of COPD among diabetes mellitus patients was significantly higher in hospital-based studies (36%; 95% CI: 26%–46%; 2 studies) than in community-based studies (15%; 95% CI: 12%–19%; 8 studies; I^2^ = 99.43%) (**Supplementary Figure 7**). The difference between study-setting subgroups was statistically significant (p<0.001).

In analysis restricted to studies involving patients specifically diagnosed with type 2 diabetes mellitus, 5 studies contributed prevalence data. The pooled prevalence of COPD among patients with type 2 diabetes mellitus was 19% (95% CI: 11%–28%) (**Supplementary Figure 8**). Considerable heterogeneity was observed across these studies (I^2^ = 94.77%; p<0.001).

### Certainty of evidence

Using GRADE, the certainty of evidence for the bidirectional comparative associations was judged to be very low. Although COPD was significantly associated with higher odds of diabetes mellitus in the overall analysis and in the adjusted-effect analysis, certainty was downgraded because the evidence was derived predominantly from observational studies, included studies with moderate or high risk of bias, used heterogeneous COPD and diabetes definitions, and showed substantial unexplained heterogeneity. The prediction interval for the overall COPD-to-diabetes association also included the null value, suggesting that the magnitude of association may vary across settings. Similarly, the certainty of evidence for diabetes mellitus as a risk factor for COPD was judged to be very low, despite a statistically significant pooled estimate, because of substantial heterogeneity, residual confounding, variation across study designs, and a prediction interval crossing the null. There was no statistical evidence of small-study effects in the main bidirectional analyses, but this did not materially increase certainty.

The certainty of evidence for the burden estimates was also judged to be very low. Although the pooled prevalence estimates suggested a considerable burden of diabetes mellitus among COPD patients and COPD among diabetes mellitus patients, confidence in these pooled estimates was limited by extreme between-study heterogeneity, differences in study setting and geographical region, variable diagnostic methods, and inclusion of populations with differing baseline risks. The subgroup analyses suggested possible variation by setting, particularly for the prevalence of COPD among diabetes mellitus patients, but heterogeneity remained substantial. Therefore, the pooled prevalence estimates should be interpreted as approximate summary estimates of burden rather than precise universal prevalence values.

## Discussion

This review provides comprehensive synthesis of the bidirectional relationship between COPD and diabetes mellitus. Across 31 included studies, the available observational data suggest possible bidirectional link between COPD and diabetes mellitus, though they frequently coexist as comorbidities in these populations. In COPD-to-diabetes analysis, patients with COPD exhibited higher odds of diabetes mellitus compared with non-COPD populations; however, this apparent crude association was substantially attenuated in adjusted analyses. This attenuation highlights that shared confounding factors such as age, smoking, adiposity, physical inactivity, and baseline cardiometabolic status partly drive this relationship, leaving independent metabolic risk attributable to COPD uncertain given the low quality of evidence. The analysis restricted to type 2 diabetes mellitus also showed significant association, supporting the relevance of COPD to metabolic risk specifically in relation to type 2 diabetes.

The reverse-direction analysis similarly pointed towards statistical association, with diabetes mellitus linked to an increased risk of subsequent COPD. However, analysis limited to type 2 diabetes mellitus showed positive but non-significant association, indicating that evidence for type 2 diabetes specifically as predictor of COPD is less consistent than broader diabetes mellitus analysis. The burden analyses further showed that diabetes mellitus was present in approximately 17% of patients with COPD, while COPD was also present in approximately 17% of patients with diabetes mellitus. Among type 2 diabetes mellitus populations, the pooled prevalence of COPD was 19%, and among COPD patients, the pooled prevalence of type 2 diabetes mellitus was 16%. These findings suggest that the coexistence of COPD and diabetes mellitus is clinically substantial, regardless of the direction from which the comorbidity is assessed.

When contrasted with existing meta-analytic literature, our study provides broader, bidirectional framework. Previously, systematic review by Peng et al. pooled longitudinal cohort data to demonstrate that baseline COPD significantly increased the risk of incident type 2 diabetes (12). While our pooled adjusted effect size for the forward direction aligns closely with their findings, our analysis expands on this by demonstrating that the association persists across cross-sectional and large administrative database designs, which Peng et al. excluded (12).

Crucially, prior systematic reviews in this domain have predominantly focused on single direction (COPD driving diabetes) or have isolated specific treatment-related metabolic complications, such as the meta-analyses by Golubic et al. (9) and Kholis et al. (10), which quantified new-onset hyperglycaemia purely from systemic and inhaled corticosteroid exposures. Our work directly fills major gap in literature by meta-analysing the reverse axis. By explicitly contrasting observational risk estimates against genetic Mendelian randomization framework, which uniquely demonstrated an inverse association, this review showed clear divergence that was unaddressed by previous reviews (9, 10). This indicates that the apparent risk of COPD in diabetic individuals observed in large cohorts like the UK Biobank may be driven by residual confounding or surveillance bias rather than true shared genetic liability (13).

The prevalence estimates in this review also support the clinical impression that COPD and diabetes mellitus frequently cluster in real-world populations. However, the very high heterogeneity observed in prevalence analyses suggests that a single pooled estimate should not be interpreted as a universal prevalence value. Differences in diagnostic criteria, population source, age structure, smoking burden, obesity prevalence, healthcare access, and ascertainment intensity probably contributed to the wide variation across studies. For example, hospital-based populations and acute exacerbation cohorts may include patients with more severe disease and higher cumulative corticosteroid exposure, whereas community-based cohorts may capture milder or undiagnosed disease. Therefore, the pooled prevalence estimates are best interpreted as summary indicators of comorbidity burden rather than fixed estimates applicable to every clinical setting.

Given that I (2) statistics across several of our primary and secondary syntheses exceeded 90%, resulting pooled point estimates must not be interpreted as monolithic, universal effect sizes. In presence of mathematical heterogeneity of this magnitude, fixed point estimate represents weighted average of distribution of effects rather than single underlying truth. Clinical and methodological diversity ranging from differences in spirometric diagnostic cutoffs to variable adjustments for critical confounders like systemic corticosteroid exposure means that true clinical relationship operates along spectrum. Consequently, calculated 95% prediction intervals are vital for proper interpretation, as they accurately depict true real-world variation and underscore expectation that future distinct clinical populations will display varying magnitudes of association.

The bidirectional associations observed are supported by distinct clinical and biological mechanisms, though their relative contributions remain difficult to isolate due to very low evidence certainty. In the forward direction, chronic systemic inflammation (driven by TNF-α, IL-6, and CRP), hypoxemia, and skeletal muscle dysfunction promote peripheral insulin resistance (47–51). This pathobiology is further compounded by treatment artifacts; as established by recent steroid-specific meta-analyses, cumulative exposure to systemic glucocorticoids during acute exacerbations sharply accelerates new-onset dysglycaemia (9). Conversely, the reverse relationship may be mediated by chronic hyperglycaemia, which induces pulmonary microangiopathy, accelerates advanced glycation end-product accumulation, and alters extracellular matrix turnover, thereby compromising lung elasticity (6, 7). The fact that COPD prevalence was significantly higher in hospital-based diabetes cohorts (36%) compared to community settings (15%) strongly implies that these biological pathways interact most aggressively in severe phenotypes, though intensive clinical surveillance in tertiary care settings remains a potential confounding factor (52, 53).

The higher COPD prevalence among hospital-based diabetes populations may reflect both biological severity and ascertainment effects. Hospitalized or specialist-treated patients are more likely to have multiple comorbidities, greater systemic inflammation, more intensive diagnostic evaluation, and higher cumulative exposure to medications that affect respiratory or metabolic status (54). Similarly, COPD patients in hospital-based cohorts may have more severe airflow limitation, frequent exacerbations, or greater corticosteroid exposure, all of which may increase the probability of detecting diabetes. Therefore, part of the observed setting-specific variation may reflect true differences in disease burden, while part may reflect detection bias and differences in clinical surveillance (55).

This review has several strengths. First, it examined the relationship between COPD and diabetes mellitus bidirectionally, rather than focusing only on diabetes among COPD patients or COPD among diabetes patients. This approach provides a more complete understanding of the clinical overlap between the two conditions. Second, the review included a large number of participants across diverse geographical regions, study designs, and healthcare settings. Third, separate analyses were performed for comparative associations, adjusted estimates, type 2 diabetes mellitus-specific data, and prevalence burden, allowing more in-depth interpretation of the evidence. Fourth, subgroup analyses, leave-one-out sensitivity analyses, meta-regression, publication-bias assessment, and GRADE certainty assessment were incorporated, improving the transparency and interpretability of the findings.

However, several limitations should be acknowledged. The certainty of evidence was very low for the main outcomes, primarily because the evidence base was largely observational and affected by substantial heterogeneity, variable risk of bias, and possible residual confounding. COPD and diabetes mellitus definitions varied considerably across studies, including spirometry-based definitions, administrative codes, physician diagnosis, self-report, medication use, and laboratory-based criteria.

Another limitation is that adjusted estimates differed across studies in the covariates included. Some studies adjusted for smoking, age, sex, and body mass index, whereas others had limited adjustment for key confounders such as corticosteroid exposure, socioeconomic status, air pollution, physical activity, COPD severity, and cardiovascular comorbidity. The very high heterogeneity in prevalence analyses also limits the precision of pooled burden estimates. Finally, publication-bias tests were only possible for analyses with sufficient numbers of studies and may have been underpowered, especially in the presence of high heterogeneity.

While our findings point toward an association between COPD and diabetes mellitus, ‘very low’ certainty of overall evidence base dictates that any definitive clinical conclusions remain preliminary. Observed patterns support, rather than firmly mandate, the concept of integrated cardiometabolic assessment in patients with COPD, particularly among older adults or those with prolonged corticosteroid exposure. Given high risk of residual confounding and methodological heterogeneity across the primary literature, routine screening of glycaemic status via fasting plasma glucose or glycated haemoglobin should be considered as a cautious, exploratory measure rather than an absolute clinical standard. Similarly, while clinicians managing diabetes mellitus should remain observant of unexplained chronic respiratory symptoms, diagnostic pathways like spirometry should be applied selectively based on individual risk profiles (e.g., extensive smoking history) rather than generalized protocols.

The findings also suggest more integrated chronic disease management model. Pulmonary rehabilitation, smoking cessation, physical activity promotion, nutritional optimization, weight management, vaccination, and careful corticosteroid stewardship may benefit both respiratory and metabolic outcomes. During COPD exacerbations, glucose monitoring may be particularly important in patients receiving systemic corticosteroids, even among those without previously diagnosed diabetes. In patients with established diabetes mellitus, respiratory symptoms should not be attributed solely to obesity, deconditioning, or cardiovascular disease without considering COPD as a potential contributor. Coordinated care between pulmonology, primary care, endocrinology, and rehabilitation services may help reduce missed diagnoses and improve long-term outcomes.

Future research should prioritize prospective cohort studies with standardized and validated definitions of both COPD and diabetes mellitus. COPD should ideally be confirmed using post-bronchodilator spirometry, while diabetes mellitus should be defined using laboratory criteria, medication use, or validated clinical records. Studies should distinguish type 1 diabetes, type 2 diabetes, steroid-induced diabetes, and unspecified diabetes wherever possible. Future analyses should also account for COPD severity, exacerbation frequency, smoking intensity, body mass index, physical activity, corticosteroid exposure, air pollution, socioeconomic factors, and comorbid cardiovascular disease.

## Conclusion

This meta-analysis suggests bidirectional association between COPD and diabetes mellitus, with COPD associated with higher odds of diabetes mellitus and diabetes mellitus associated with higher risk of COPD. Pooled burden estimates indicate that approximately one in six patients with either condition may have the other comorbidity, underscoring the clinical relevance of integrated assessment. However, the certainty of evidence was very low because of the observational nature of the evidence, substantial heterogeneity, variable diagnostic definitions, and residual confounding. These findings support greater clinical awareness of COPD–diabetes overlap, while emphasizing the need for high-quality longitudinal and mechanistic studies to clarify causality, identify high-risk phenotypes, and guide integrated prevention and management strategies.

## Data Availability

The data that supports the finding of this study are available from the corresponding author upon reasonable request.

## Supplementary material

Supplementary Figure 1 Leave-one-out sensitivity analysis for diabetes mellitus risk among COPD patients. Sensitivity analysis evaluating the influence of each individual study on the overall pooled association between COPD and diabetes mellitus risk.

Supplementary Figure 2 Funnel plot for publication bias in the analysis of diabetes mellitus risk among COPD patients. Assessment of funnel-plot asymmetry and small-study effects for the overall COPD-to-diabetes association; Egger’s regression test showed no statistically significant evidence of small-study effects.

Supplementary Figure 3 Leave-one-out sensitivity analysis for COPD risk among diabetes mellitus patients. Sensitivity analysis evaluating the influence of each individual study on the overall pooled association between diabetes mellitus and COPD risk.

Supplementary Figure 4 Funnel plot for publication bias in the analysis of COPD risk among diabetes mellitus patients. Assessment of funnel-plot asymmetry and small-study effects for the overall diabetes-to-COPD association; Egger’s regression test showed no statistically significant evidence of small-study effects.

Supplementary Figure 5 Forest plot of the pooled prevalence of COPD among patients with diabetes mellitus. Random-effects prevalence meta-analysis estimating the burden of COPD in diabetes mellitus populations.

Supplementary Figure 6 Subgroup analysis of COPD prevalence among diabetes mellitus patients by geographical region. Random-effects subgroup meta-analysis of COPD burden among diabetes mellitus patients stratified by Asia and Europe.

Supplementary Figure 7 Subgroup analysis of COPD prevalence among diabetes mellitus patients by study setting. Random-effects subgroup meta-analysis comparing COPD prevalence between community-based and hospital-based diabetes mellitus populations.

Supplementary Figure 8 Forest plot of the pooled prevalence of COPD among patients with type 2 diabetes mellitus. Random-effects prevalence meta-analysis restricted to studies involving patients with type 2 diabetes mellitus.
