# Supplementary material for: Chronic obstructive pulmonary disease and diabetes mellitus: a bidirectional meta-analysis of risk and comorbidity burden
Source: Front Endocrinol (Lausanne). 2026 Aug 4;17:1912659. doi: 10.3389/fendo.2026.1912659 (PMC13481201; doi:10.3389/fendo.2026.1912659)
Supplement: Supplementary file 1 [file DataSheet1.pdf]

Supplementary File

Search Strategy

PubMed:

(("Pulmonary Disease, Chronic Obstructive"[Mesh] OR "Lung Diseases, Obstructive"[Mesh] OR "Bronchitis, Chronic"[Mesh] OR "Pulmonary Emphysema"[Mesh] OR "chronic obstructive pulmonary disease"[tiab] OR "chronic obstructive pulmonary diseases"[tiab] OR "chronic obstructive lung disease"[tiab] OR "chronic obstructive lung diseases"[tiab] OR "chronic obstructive airway disease"[tiab] OR "chronic obstructive airway diseases"[tiab] OR "chronic obstructive airways disease"[tiab] OR "chronic obstructive respiratory disease"[tiab] OR "chronic obstructive respiratory diseases"[tiab] OR "chronic obstructive pulmonary disorder"[tiab] OR "chronic obstructive pulmonary disorders"[tiab] OR "chronic obstructive lung disorder"[tiab] OR "chronic obstructive lung disorders"[tiab] OR "obstructive pulmonary disease"[tiab] OR "obstructive pulmonary diseases"[tiab] OR "obstructive lung disease"[tiab] OR "obstructive lung diseases"[tiab] OR "obstructive airway disease"[tiab] OR "obstructive airway diseases"[tiab] OR "obstructive airways disease"[tiab] OR "obstructive respiratory disease"[tiab] OR "obstructive respiratory diseases"[tiab] OR COPD[tiab] OR COAD[tiab] OR COLD[tiab] OR AECOPD[tiab] OR ECOPD[tiab] OR "acute exacerbation of chronic obstructive pulmonary disease"[tiab] OR "acute exacerbation of COPD"[tiab] OR "COPD exacerbation"[tiab] OR "COPD exacerbations"[tiab] OR "exacerbated COPD"[tiab] OR "stable COPD"[tiab] OR "severe COPD"[tiab] OR "moderate COPD"[tiab] OR "mild COPD"[tiab] OR "advanced COPD"[tiab] OR "COPD patient"[tiab] OR "COPD patients"[tiab] OR "COPD population"[tiab] OR "COPD cohort"[tiab] OR "Global Initiative for Chronic Obstructive Lung Disease"[tiab] OR "GOLD criteria"[tiab] OR "GOLD-defined COPD"[tiab] OR "GOLD stage"[tiab] OR "GOLD stages"[tiab] OR "chronic airflow

obstruction"[tiab] OR "chronic airflow limitation"[tiab] OR "persistent airflow limitation"[tiab] OR "persistent airflow obstruction"[tiab] OR "fixed airflow obstruction"[tiab] OR "fixed airflow limitation"[tiab] OR "airflow obstruction"[tiab] OR "airflow limitation"[tiab] OR "airway obstruction"[tiab] OR "airways obstruction"[tiab] OR "bronchial obstruction"[tiab] OR "spirometric obstruction"[tiab] OR "spirometric airflow obstruction"[tiab] OR "obstructive spirometry"[tiab] OR "obstructive spirometric pattern"[tiab] OR "obstructive ventilatory defect"[tiab] OR "obstructive ventilatory impairment"[tiab] OR "post-bronchodilator obstruction"[tiab] OR "post bronchodilator obstruction"[tiab] OR "post-bronchodilator airflow obstruction"[tiab] OR "post bronchodilator airflow obstruction"[tiab] OR "FEV1/FVC"[tiab] OR "FEV1 FVC"[tiab] OR "FEV1/FVC ratio"[tiab] OR "forced expiratory volume/forced vital capacity"[tiab] OR "forced expiratory volume in one second/forced vital capacity"[tiab] OR "chronic bronchitis"[tiab] OR "chronic bronchitides"[tiab] OR emphysema[tiab] OR emphysematous[tiab] OR "pulmonary emphysema"[tiab] OR "lung emphysema"[tiab]) AND ("Diabetes Mellitus"[Mesh] OR "Diabetes Mellitus, Type 2"[Mesh] OR "Diabetes Mellitus, Type 1"[Mesh] OR "Diabetes Complications"[Mesh] OR "Prediabetic State"[Mesh] OR "Hyperglycemia"[Mesh] OR "Blood Glucose"[Mesh] OR "Glucose Intolerance"[Mesh] OR "Insulin Resistance"[Mesh] OR "Hemoglobin A, Glycosylated"[Mesh] OR "Hypoglycemic Agents"[Mesh] OR "diabetes mellitus"[tiab] OR diabetes[tiab] OR diabetic*[tiab] OR diabet*[tiab] OR "type 2 diabetes"[tiab] OR "type-2 diabetes"[tiab] OR "type II diabetes"[tiab] OR "type-II diabetes"[tiab] OR "type 2 diabetes mellitus"[tiab] OR "type-2 diabetes mellitus"[tiab] OR "type II diabetes mellitus"[tiab] OR "type-II diabetes mellitus"[tiab] OR "T2DM"[tiab] OR "T2D"[tiab] OR "type 2 DM"[tiab] OR "type II DM"[tiab] OR "non insulin dependent diabetes"[tiab] OR "non-insulin dependent diabetes"[tiab] OR "noninsulin dependent diabetes"[tiab] OR "non insulin dependent diabetes

mellitus"[tiab] OR "non-insulin dependent diabetes mellitus"[tiab] OR "noninsulin dependent diabetes mellitus"[tiab] OR NIDDM[tiab] OR "adult onset diabetes"[tiab] OR "adult-onset diabetes"[tiab] OR "maturity onset diabetes"[tiab] OR "maturity-onset diabetes"[tiab] OR "type 1 diabetes"[tiab] OR "type-1 diabetes"[tiab] OR "type I diabetes"[tiab] OR "type-I diabetes"[tiab] OR "type 1 diabetes mellitus"[tiab] OR "type-1 diabetes mellitus"[tiab] OR "type I diabetes mellitus"[tiab] OR "type-I diabetes mellitus"[tiab] OR "T1DM"[tiab] OR "T1D"[tiab] OR "type 1 DM"[tiab] OR "type I DM"[tiab] OR "insulin dependent diabetes"[tiab] OR "insulin-dependent diabetes"[tiab] OR "insulin dependent diabetes mellitus"[tiab] OR "insulin-dependent diabetes mellitus"[tiab] OR IDDM[tiab] OR "new onset diabetes"[tiab] OR "new-onset diabetes"[tiab] OR "incident diabetes"[tiab] OR "prevalent diabetes"[tiab] OR "undiagnosed diabetes"[tiab] OR hyperglycemia[tiab] OR hyperglycaemia[tiab] OR hyperglycemic[tiab] OR hyperglycaemic[tiab] OR hyperglycem*[tiab] OR hyperglycaem*[tiab] OR glycemic[tiab] OR glycaemic[tiab] OR glycem*[tiab] OR glycaem*[tiab] OR "glycemic status"[tiab] OR "glycaemic status"[tiab] OR "glycemic control"[tiab] OR "glycaemic control"[tiab] OR "glucose control"[tiab] OR "poor glycemic control"[tiab] OR "poor glycaemic control"[tiab] OR "blood glucose"[tiab] OR "blood sugar"[tiab] OR "plasma glucose"[tiab] OR "serum glucose"[tiab] OR "fasting glucose"[tiab] OR "fasting plasma glucose"[tiab] OR "fasting blood glucose"[tiab] OR "random blood glucose"[tiab] OR "postprandial glucose"[tiab] OR "oral glucose tolerance"[tiab] OR OGTT[tiab] OR HbA1c[tiab] OR "Hb A1c"[tiab] OR A1c[tiab] OR "hemoglobin A1c"[tiab] OR "haemoglobin A1c"[tiab] OR "glycated hemoglobin"[tiab] OR "glycated haemoglobin"[tiab] OR "glycosylated hemoglobin"[tiab] OR "glycosylated haemoglobin"[tiab] OR "glucose intolerance"[tiab] OR "impaired glucose tolerance"[tiab] OR "impaired fasting glucose"[tiab] OR "impaired glucose regulation"[tiab] OR "impaired glucose metabolism"[tiab] OR IFG[tiab] OR IGT[tiab] OR prediabetes[tiab] OR

prediabetic[tiab] OR "pre diabetes"[tiab] OR "pre-diabetes"[tiab] OR "pre diabetic"[tiab] OR "pre-diabetic"[tiab] OR "prediabetic state"[tiab] OR "insulin resistance"[tiab] OR "insulin resistant"[tiab] OR "insulin sensitivity"[tiab] OR "impaired insulin sensitivity"[tiab] OR "reduced insulin sensitivity"[tiab] OR antidiabetic[tiab] OR anti-diabetic[tiab] OR "anti diabetic"[tiab] OR "glucose lowering"[tiab] OR "glucose-lowering"[tiab] OR "hypoglycemic agent"[tiab] OR "hypoglycemic agents"[tiab] OR "hypoglycaemic agent"[tiab] OR "hypoglycaemic agents"[tiab]))

EMBASE:

('chronic obstructive lung disease'/exp OR 'chronic obstructive pulmonary disease'/exp OR 'chronic bronchitis'/exp OR 'lung emphysema'/exp OR 'pulmonary emphysema'/exp OR 'emphysema'/exp OR 'obstructive airway disease'/exp OR 'obstructive lung disease'/exp OR 'airway obstruction'/exp OR 'airflow limitation'/exp OR 'chronic obstructive pulmonary disease':ti,ab,kw OR 'chronic obstructive pulmonary diseases':ti,ab,kw OR 'chronic obstructive lung disease':ti,ab,kw OR 'chronic obstructive lung diseases':ti,ab,kw OR 'chronic obstructive airway disease':ti,ab,kw OR 'chronic obstructive airway diseases':ti,ab,kw OR 'chronic obstructive airways disease':ti,ab,kw OR 'chronic obstructive respiratory disease':ti,ab,kw OR 'chronic obstructive respiratory diseases':ti,ab,kw OR 'chronic obstructive pulmonary disorder':ti,ab,kw OR 'chronic obstructive pulmonary disorders':ti,ab,kw OR 'chronic obstructive lung disorder':ti,ab,kw OR 'chronic obstructive lung disorders':ti,ab,kw OR 'obstructive pulmonary disease':ti,ab,kw OR 'obstructive pulmonary diseases':ti,ab,kw OR 'obstructive lung disease':ti,ab,kw OR 'obstructive lung diseases':ti,ab,kw OR 'obstructive airway disease':ti,ab,kw OR 'obstructive airway diseases':ti,ab,kw OR 'obstructive airways disease':ti,ab,kw OR 'obstructive respiratory disease':ti,ab,kw OR 'obstructive respiratory diseases':ti,ab,kw OR copd:ti,ab,kw OR

coad:ti,ab,kw OR cold:ti,ab,kw OR aecopd:ti,ab,kw OR ecopd:ti,ab,kw OR 'acute exacerbation of chronic obstructive pulmonary disease':ti,ab,kw OR 'acute exacerbation of COPD':ti,ab,kw OR 'acute exacerbations of COPD':ti,ab,kw OR 'COPD exacerbation':ti,ab,kw OR 'COPD exacerbations':ti,ab,kw OR 'exacerbated COPD':ti,ab,kw OR 'stable COPD':ti,ab,kw OR 'severe COPD':ti,ab,kw OR 'moderate COPD':ti,ab,kw OR 'mild COPD':ti,ab,kw OR 'advanced COPD':ti,ab,kw OR 'COPD patient':ti,ab,kw OR 'COPD patients':ti,ab,kw OR 'COPD population':ti,ab,kw OR 'COPD cohort':ti,ab,kw OR 'COPD subjects':ti,ab,kw OR 'COPD cases':ti,ab,kw OR 'Global Initiative for Chronic Obstructive Lung Disease':ti,ab,kw OR 'GOLD criteria':ti,ab,kw OR 'GOLD-defined COPD':ti,ab,kw OR 'GOLD stage':ti,ab,kw OR 'GOLD stages':ti,ab,kw OR 'GOLD classification':ti,ab,kw OR 'chronic airflow obstruction':ti,ab,kw OR 'chronic airflow limitation':ti,ab,kw OR 'persistent airflow limitation':ti,ab,kw OR 'persistent airflow obstruction':ti,ab,kw OR 'fixed airflow obstruction':ti,ab,kw OR 'fixed airflow limitation':ti,ab,kw OR 'airflow obstruction':ti,ab,kw OR 'airflow limitation':ti,ab,kw OR 'airway obstruction':ti,ab,kw OR 'airways obstruction':ti,ab,kw OR 'bronchial obstruction':ti,ab,kw OR 'spirometric obstruction':ti,ab,kw OR 'spirometric airflow obstruction':ti,ab,kw OR 'obstructive spirometry':ti,ab,kw OR 'obstructive spirometric pattern':ti,ab,kw OR 'obstructive ventilatory defect':ti,ab,kw OR 'obstructive ventilatory impairment':ti,ab,kw OR 'post-bronchodilator obstruction':ti,ab,kw OR 'post bronchodilator obstruction':ti,ab,kw OR 'post-bronchodilator airflow obstruction':ti,ab,kw OR 'post bronchodilator airflow obstruction':ti,ab,kw OR 'FEV1/FVC':ti,ab,kw OR 'FEV1 FVC':ti,ab,kw OR 'FEV1/FVC ratio':ti,ab,kw OR 'forced expiratory volume/forced vital capacity':ti,ab,kw OR 'forced expiratory volume in one second/forced vital capacity':ti,ab,kw OR 'forced expiratory volume in 1 second/forced vital capacity':ti,ab,kw OR 'chronic bronchitis':ti,ab,kw OR 'chronic bronchitides':ti,ab,kw OR emphysema:ti,ab,kw OR emphysematous:ti,ab,kw OR 'pulmonary emphysema':ti,ab,kw OR

'lung emphysema':ti,ab,kw) AND ('diabetes mellitus'/exp OR 'non insulin dependent diabetes mellitus'/exp OR 'insulin dependent diabetes mellitus'/exp OR 'type 2 diabetes mellitus'/exp OR 'type 1 diabetes mellitus'/exp OR 'diabetes complication'/exp OR 'diabetic complication'/exp OR 'prediabetes'/exp OR 'hyperglycemia'/exp OR 'blood glucose'/exp OR 'glucose intolerance'/exp OR 'impaired glucose tolerance'/exp OR 'insulin resistance'/exp OR 'glycosylated hemoglobin'/exp OR 'hemoglobin A1c'/exp OR 'hypoglycemic agent'/exp OR 'antidiabetic agent'/exp OR 'diabetes mellitus':ti,ab,kw OR diabetes:ti,ab,kw OR diabetic*:ti,ab,kw OR diabet*:ti,ab,kw OR 'type 2 diabetes':ti,ab,kw OR 'type-2 diabetes':ti,ab,kw OR 'type II diabetes':ti,ab,kw OR 'type-II diabetes':ti,ab,kw OR 'type 2 diabetes mellitus':ti,ab,kw OR 'type-2 diabetes mellitus':ti,ab,kw OR 'type II diabetes mellitus':ti,ab,kw OR 'type-II diabetes mellitus':ti,ab,kw OR t2dm:ti,ab,kw OR t2d:ti,ab,kw OR 'type 2 DM':ti,ab,kw OR 'type II DM':ti,ab,kw OR 'non insulin dependent diabetes':ti,ab,kw OR 'non-insulin dependent diabetes':ti,ab,kw OR 'noninsulin dependent diabetes':ti,ab,kw OR 'non insulin dependent diabetes mellitus':ti,ab,kw OR 'non-insulin dependent diabetes mellitus':ti,ab,kw OR 'noninsulin dependent diabetes mellitus':ti,ab,kw OR niddm:ti,ab,kw OR 'adult onset diabetes':ti,ab,kw OR 'adult-onset diabetes':ti,ab,kw OR 'maturity onset diabetes':ti,ab,kw OR 'maturity-onset diabetes':ti,ab,kw OR 'type 1 diabetes':ti,ab,kw OR 'type-1 diabetes':ti,ab,kw OR 'type I diabetes':ti,ab,kw OR 'type-I diabetes':ti,ab,kw OR 'type 1 diabetes mellitus':ti,ab,kw OR 'type-1 diabetes mellitus':ti,ab,kw OR 'type I diabetes mellitus':ti,ab,kw OR 'type-I diabetes mellitus':ti,ab,kw OR t1dm:ti,ab,kw OR t1d:ti,ab,kw OR 'type 1 DM':ti,ab,kw OR 'type I DM':ti,ab,kw OR 'insulin dependent diabetes':ti,ab,kw OR 'insulin-dependent diabetes':ti,ab,kw OR 'insulin dependent diabetes mellitus':ti,ab,kw OR 'insulin-dependent diabetes mellitus':ti,ab,kw OR iddm:ti,ab,kw OR 'new onset diabetes':ti,ab,kw OR 'new-onset diabetes':ti,ab,kw OR 'incident diabetes':ti,ab,kw OR 'prevalent diabetes':ti,ab,kw OR 'undiagnosed diabetes':ti,ab,kw OR

hyperglycemia:ti,ab,kw OR hyperglycaemia:ti,ab,kw OR hyperglycemic:ti,ab,kw OR hyperglycaemic:ti,ab,kw OR hyperglycem*:ti,ab,kw OR hyperglycaem*:ti,ab,kw OR glycemic:ti,ab,kw OR glycaemic:ti,ab,kw OR glycem*:ti,ab,kw OR glycaem*:ti,ab,kw OR 'glycemic status':ti,ab,kw OR 'glycaemic status':ti,ab,kw OR 'glycemic control':ti,ab,kw OR 'glycaemic control':ti,ab,kw OR 'glucose control':ti,ab,kw OR 'poor glycemic control':ti,ab,kw OR 'poor glycaemic control':ti,ab,kw OR 'blood glucose':ti,ab,kw OR 'blood sugar':ti,ab,kw OR 'plasma glucose':ti,ab,kw OR 'serum glucose':ti,ab,kw OR 'fasting glucose':ti,ab,kw OR 'fasting plasma glucose':ti,ab,kw OR 'fasting blood glucose':ti,ab,kw OR 'random blood glucose':ti,ab,kw OR 'postprandial glucose':ti,ab,kw OR 'oral glucose tolerance':ti,ab,kw OR ogtt:ti,ab,kw OR hba1c:ti,ab,kw OR 'Hb A1c':ti,ab,kw OR a1c:ti,ab,kw OR 'hemoglobin A1c':ti,ab,kw OR 'haemoglobin A1c':ti,ab,kw OR 'glycated hemoglobin':ti,ab,kw OR 'glycated haemoglobin':ti,ab,kw OR 'glycosylated hemoglobin':ti,ab,kw OR 'glycosylated haemoglobin':ti,ab,kw OR 'glucose intolerance':ti,ab,kw OR 'impaired glucose tolerance':ti,ab,kw OR 'impaired fasting glucose':ti,ab,kw OR 'impaired glucose regulation':ti,ab,kw OR 'impaired glucose metabolism':ti,ab,kw OR ifg:ti,ab,kw OR igt:ti,ab,kw OR prediabetes:ti,ab,kw OR prediabetic:ti,ab,kw OR 'pre diabetes':ti,ab,kw OR 'pre-diabetes':ti,ab,kw OR 'pre diabetic':ti,ab,kw OR 'pre-diabetic':ti,ab,kw OR 'prediabetic state':ti,ab,kw OR 'insulin resistance':ti,ab,kw OR 'insulin resistant':ti,ab,kw OR 'insulin sensitivity':ti,ab,kw OR 'impaired insulin sensitivity':ti,ab,kw OR 'reduced insulin sensitivity':ti,ab,kw OR antidiabetic:ti,ab,kw OR anti-diabetic:ti,ab,kw OR 'anti diabetic':ti,ab,kw OR 'glucose lowering':ti,ab,kw OR 'glucose-lowering':ti,ab,kw OR 'hypoglycemic agent':ti,ab,kw OR 'hypoglycemic agents':ti,ab,kw OR 'hypoglycaemic agent':ti,ab,kw OR 'hypoglycaemic agents':ti,ab,kw)

Scopus:

TITLE-ABS-KEY("chronic obstructive pulmonary disease" OR "chronic obstructive pulmonary diseases" OR "chronic obstructive lung disease" OR "chronic obstructive lung diseases" OR "chronic obstructive airway disease" OR "chronic obstructive airway diseases" OR "chronic obstructive airways disease" OR "chronic obstructive respiratory disease" OR "chronic obstructive respiratory diseases" OR "chronic obstructive pulmonary disorder" OR "chronic obstructive pulmonary disorders" OR "chronic obstructive lung disorder" OR "chronic obstructive lung disorders" OR "obstructive pulmonary disease" OR "obstructive pulmonary diseases" OR "obstructive lung disease" OR "obstructive lung diseases" OR "obstructive airway disease" OR "obstructive airway diseases" OR "obstructive airways disease" OR "obstructive respiratory disease" OR "obstructive respiratory diseases" OR COPD OR COAD OR COLD OR AECOPD OR ECOPD OR "acute exacerbation of chronic obstructive pulmonary disease" OR "acute exacerbation of COPD" OR "acute exacerbations of COPD" OR "COPD exacerbation" OR "COPD exacerbations" OR "exacerbated COPD" OR "stable COPD" OR "severe COPD" OR "moderate COPD" OR "mild COPD" OR "advanced COPD" OR "COPD patient" OR "COPD patients" OR "COPD population" OR "COPD cohort" OR "COPD subjects" OR "COPD cases" OR "Global Initiative for Chronic Obstructive Lung Disease" OR "GOLD criteria" OR "GOLD-defined COPD" OR "GOLD stage" OR "GOLD stages" OR "GOLD classification" OR "chronic airflow obstruction" OR "chronic airflow limitation" OR "persistent airflow limitation" OR "persistent airflow obstruction" OR "fixed airflow obstruction" OR "fixed airflow limitation" OR "airflow obstruction" OR "airflow limitation" OR "airway obstruction" OR "airways obstruction" OR "bronchial obstruction" OR "spirometric obstruction" OR "spirometric airflow obstruction" OR "obstructive spirometry" OR "obstructive spirometric pattern" OR "obstructive ventilatory defect" OR "obstructive ventilatory impairment" OR "post-bronchodilator

obstruction" OR "post bronchodilator obstruction" OR "post-bronchodilator airflow obstruction" OR "post bronchodilator airflow obstruction" OR "FEV1/FVC" OR "FEV1 FVC" OR "FEV1/FVC ratio" OR "forced expiratory volume/forced vital capacity" OR "forced expiratory volume in one second/forced vital capacity" OR "forced expiratory volume in 1 second/forced vital capacity" OR "chronic bronchitis" OR "chronic bronchitides" OR emphysema OR emphysematous OR "pulmonary emphysema" OR "lung emphysema") AND TITLE-ABS-KEY("diabetes mellitus" OR diabetes OR diabetic* OR diabet* OR "type 2 diabetes" OR "type-2 diabetes" OR "type II diabetes" OR "type-II diabetes" OR "type 2 diabetes mellitus" OR "type-2 diabetes mellitus" OR "type II diabetes mellitus" OR "type-II diabetes mellitus" OR T2DM OR T2D OR "type 2 DM" OR "type II DM" OR "non insulin dependent diabetes" OR "non-insulin dependent diabetes" OR "noninsulin dependent diabetes" OR "non insulin dependent diabetes mellitus" OR "non-insulin dependent diabetes mellitus" OR "noninsulin dependent diabetes mellitus" OR NIDDM OR "adult onset diabetes" OR "adult-onset diabetes" OR "maturity onset diabetes" OR "maturity-onset diabetes" OR "type 1 diabetes" OR "type-1 diabetes" OR "type I diabetes" OR "type-I diabetes" OR "type 1 diabetes mellitus" OR "type-1 diabetes mellitus" OR "type I diabetes mellitus" OR "type-I diabetes mellitus" OR T1DM OR T1D OR "type 1 DM" OR "type I DM" OR "insulin dependent diabetes" OR "insulin-dependent diabetes" OR "insulin dependent diabetes mellitus" OR "insulin-dependent diabetes mellitus" OR IDDM OR "new onset diabetes" OR "new-onset diabetes" OR "incident diabetes" OR "prevalent diabetes" OR "undiagnosed diabetes" OR hyperglycemia OR hyperglycaemia OR hyperglycemic OR hyperglycaemic OR hyperglycem* OR hyperglycaem* OR glycemic OR glycaemic OR glycem* OR glycaem* OR "glycemic status" OR "glycaemic status" OR "glycemic control" OR "glycaemic control" OR "glucose control" OR "poor glycemic control" OR "poor glycaemic control" OR "blood glucose" OR "blood sugar" OR "plasma glucose" OR "serum

glucose" OR "fasting glucose" OR "fasting plasma glucose" OR "fasting blood glucose" OR "random blood glucose" OR "postprandial glucose" OR "oral glucose tolerance" OR OGTT OR HbA1c OR "Hb A1c" OR A1c OR "hemoglobin A1c" OR "haemoglobin A1c" OR "glycated hemoglobin" OR "glycated haemoglobin" OR "glycosylated hemoglobin" OR "glycosylated haemoglobin" OR "glucose intolerance" OR "impaired glucose tolerance" OR "impaired fasting glucose" OR "impaired glucose regulation" OR "impaired glucose metabolism" OR IFG OR IGT OR prediabetes OR prediabetic OR "pre diabetes" OR "pre-diabetes" OR "pre diabetic" OR "pre-diabetic" OR "prediabetic state" OR "insulin resistance" OR "insulin resistant" OR "insulin sensitivity" OR "impaired insulin sensitivity" OR "reduced insulin sensitivity" OR antidiabetic OR anti-diabetic OR "anti diabetic" OR "glucose lowering" OR "glucose-lowering" OR "hypoglycemic agent" OR "hypoglycemic agents" OR "hypoglycaemic agent" OR "hypoglycaemic agents") AND NOT (TITLE-ABS-KEY(animal* OR rat OR rats OR mouse OR mice OR murine OR rabbit* OR dog OR dogs OR canine OR cat OR cats OR feline OR pig OR pigs OR porcine OR sheep OR ovine OR goat* OR bovine OR cattle OR monkey* OR primate* OR rodent* OR zebrafish OR "in vitro" OR "cell line" OR "cell lines") AND NOT TITLE-ABS-KEY(human* OR patient* OR participant* OR subject* OR adult* OR men OR women OR male* OR female* OR cohort* OR population*))

Web of Science:

TS=("chronic obstructive pulmonary disease" OR "chronic obstructive pulmonary diseases" OR "chronic obstructive lung disease" OR "chronic obstructive lung diseases" OR "chronic obstructive airway disease" OR "chronic obstructive airway diseases" OR "chronic obstructive airways disease" OR "chronic obstructive respiratory disease" OR "chronic obstructive respiratory diseases" OR "chronic obstructive pulmonary disorder" OR "chronic

obstructive pulmonary disorders" OR "chronic obstructive lung disorder" OR "chronic obstructive lung disorders" OR "obstructive pulmonary disease" OR "obstructive pulmonary diseases" OR "obstructive lung disease" OR "obstructive lung diseases" OR "obstructive airway disease" OR "obstructive airway diseases" OR "obstructive airways disease" OR "obstructive respiratory disease" OR "obstructive respiratory diseases" OR COPD OR COAD OR COLD OR AECOPD OR ECOPD OR "acute exacerbation of chronic obstructive pulmonary disease" OR "acute exacerbation of COPD" OR "acute exacerbations of COPD" OR "COPD exacerbation" OR "COPD exacerbations" OR "exacerbated COPD" OR "stable COPD" OR "severe COPD" OR "moderate COPD" OR "mild COPD" OR "advanced COPD" OR "COPD patient" OR "COPD patients" OR "COPD population" OR "COPD cohort" OR "COPD subjects" OR "COPD cases" OR "Global Initiative for Chronic Obstructive Lung Disease" OR "GOLD criteria" OR "GOLD-defined COPD" OR "GOLD stage" OR "GOLD stages" OR "GOLD classification" OR "chronic airflow obstruction" OR "chronic airflow limitation" OR "persistent airflow limitation" OR "persistent airflow obstruction" OR "fixed airflow obstruction" OR "fixed airflow limitation" OR "airflow obstruction" OR "airflow limitation" OR "airway obstruction" OR "airways obstruction" OR "bronchial obstruction" OR "spirometric obstruction" OR "spirometric airflow obstruction" OR "obstructive spirometry" OR "obstructive spirometric pattern" OR "obstructive ventilatory defect" OR "obstructive ventilatory impairment" OR "post-bronchodilator obstruction" OR "post bronchodilator obstruction" OR "post-bronchodilator airflow obstruction" OR "post bronchodilator airflow obstruction" OR "FEV1/FVC" OR "FEV1 FVC" OR "FEV1/FVC ratio" OR "forced expiratory volume/forced vital capacity" OR "forced expiratory volume in one second/forced vital capacity" OR "forced expiratory volume in 1 second/forced vital capacity" OR "chronic bronchitis" OR "chronic bronchitides" OR emphysema OR emphysematous OR "pulmonary emphysema" OR "lung emphysema") AND TS=("diabetes

mellitus" OR diabetes OR diabetic* OR diabet* OR "type 2 diabetes" OR "type-2 diabetes" OR "type II diabetes" OR "type-II diabetes" OR "type 2 diabetes mellitus" OR "type-2 diabetes mellitus" OR "type II diabetes mellitus" OR "type-II diabetes mellitus" OR T2DM OR T2D OR "type 2 DM" OR "type II DM" OR "non insulin dependent diabetes" OR "non-insulin dependent diabetes" OR "noninsulin dependent diabetes" OR "non insulin dependent diabetes mellitus" OR "non-insulin dependent diabetes mellitus" OR "noninsulin dependent diabetes mellitus" OR NIDDM OR "adult onset diabetes" OR "adult-onset diabetes" OR "maturity onset diabetes" OR "maturity-onset diabetes" OR "type 1 diabetes" OR "type-1 diabetes" OR "type I diabetes" OR "type-I diabetes" OR "type 1 diabetes mellitus" OR "type-1 diabetes mellitus" OR "type I diabetes mellitus" OR "type-I diabetes mellitus" OR T1DM OR T1D OR "type 1 DM" OR "type I DM" OR "insulin dependent diabetes" OR "insulin-dependent diabetes" OR "insulin dependent diabetes mellitus" OR "insulin-dependent diabetes mellitus" OR IDDM OR "new onset diabetes" OR "new-onset diabetes" OR "incident diabetes" OR "prevalent diabetes" OR "undiagnosed diabetes" OR hyperglycemia OR hyperglycaemia OR hyperglycemic OR hyperglycaemic OR hyperglycem* OR hyperglycaem* OR glycemic OR glycaemic OR glycem* OR glycaem* OR "glycemic status" OR "glycaemic status" OR "glycemic control" OR "glycaemic control" OR "glucose control" OR "poor glycemic control" OR "poor glycaemic control" OR "blood glucose" OR "blood sugar" OR "plasma glucose" OR "serum glucose" OR "fasting glucose" OR "fasting plasma glucose" OR "fasting blood glucose" OR "random blood glucose" OR "postprandial glucose" OR "oral glucose tolerance" OR OGTT OR HbA1c OR "Hb A1c" OR A1c OR "hemoglobin A1c" OR "haemoglobin A1c" OR "glycated hemoglobin" OR "glycated haemoglobin" OR "glycosylated hemoglobin" OR "glycosylated haemoglobin" OR "glucose intolerance" OR "impaired glucose tolerance" OR "impaired fasting glucose" OR "impaired glucose regulation" OR "impaired glucose metabolism" OR IFG OR IGT OR prediabetes OR

prediabetic OR "pre diabetes" OR "pre-diabetes" OR "pre diabetic" OR "pre-diabetic" OR "prediabetic state" OR "insulin resistance" OR "insulin resistant" OR "insulin sensitivity" OR "impaired insulin sensitivity" OR "reduced insulin sensitivity" OR antidiabetic OR anti-diabetic OR "anti diabetic" OR "glucose lowering" OR "glucose-lowering" OR "hypoglycemic agent" OR "hypoglycemic agents" OR "hypoglycaemic agent" OR "hypoglycaemic agents") NOT (TS=(animal* OR rat OR rats OR mouse OR mice OR murine OR rabbit* OR dog OR dogs OR canine OR cat OR cats OR feline OR pig OR pigs OR porcine OR sheep OR ovine OR goat* OR bovine OR cattle OR monkey* OR primate* OR rodent* OR zebrafish OR "in vitro" OR "cell line" OR "cell lines") NOT TS=(human* OR patient* OR participant* OR subject* OR adult* OR men OR women OR male* OR female* OR cohort* OR population*))

Cochrane library:

([mh "Pulmonary Disease, Chronic Obstructive"] OR [mh "Lung Diseases, Obstructive"] OR [mh "Bronchitis, Chronic"] OR [mh "Pulmonary Emphysema"] OR "chronic obstructive pulmonary disease":ti,ab,kw OR "chronic obstructive pulmonary diseases":ti,ab,kw OR "chronic obstructive lung disease":ti,ab,kw OR "chronic obstructive lung diseases":ti,ab,kw OR "chronic obstructive airway disease":ti,ab,kw OR "chronic obstructive airway diseases":ti,ab,kw OR "chronic obstructive airways disease":ti,ab,kw OR "chronic obstructive respiratory disease":ti,ab,kw OR "chronic obstructive respiratory diseases":ti,ab,kw OR "chronic obstructive pulmonary disorder":ti,ab,kw OR "chronic obstructive pulmonary disorders":ti,ab,kw OR "chronic obstructive lung disorder":ti,ab,kw OR "chronic obstructive lung disorders":ti,ab,kw OR "obstructive pulmonary disease":ti,ab,kw OR "obstructive pulmonary diseases":ti,ab,kw OR "obstructive lung disease":ti,ab,kw OR "obstructive lung diseases":ti,ab,kw OR "obstructive airway disease":ti,ab,kw OR "obstructive airway

diseases":ti,ab,kw OR "obstructive airways disease":ti,ab,kw OR "obstructive respiratory disease":ti,ab,kw OR "obstructive respiratory diseases":ti,ab,kw OR COPD:ti,ab,kw OR COAD:ti,ab,kw OR COLD:ti,ab,kw OR AECOPD:ti,ab,kw OR ECOPD:ti,ab,kw OR "acute exacerbation of chronic obstructive pulmonary disease":ti,ab,kw OR "acute exacerbation of COPD":ti,ab,kw OR "acute exacerbations of COPD":ti,ab,kw OR "COPD exacerbation":ti,ab,kw OR "COPD exacerbations":ti,ab,kw OR "exacerbated COPD":ti,ab,kw OR "stable COPD":ti,ab,kw OR "severe COPD":ti,ab,kw OR "moderate COPD":ti,ab,kw OR "mild COPD":ti,ab,kw OR "advanced COPD":ti,ab,kw OR "COPD patient":ti,ab,kw OR "COPD patients":ti,ab,kw OR "COPD population":ti,ab,kw OR "COPD cohort":ti,ab,kw OR "COPD subjects":ti,ab,kw OR "COPD cases":ti,ab,kw OR "Global Initiative for Chronic Obstructive Lung Disease":ti,ab,kw OR "GOLD criteria":ti,ab,kw OR "GOLD-defined COPD":ti,ab,kw OR "GOLD stage":ti,ab,kw OR "GOLD stages":ti,ab,kw OR "GOLD classification":ti,ab,kw OR "chronic airflow obstruction":ti,ab,kw OR "chronic airflow limitation":ti,ab,kw OR "persistent airflow limitation":ti,ab,kw OR "persistent airflow obstruction":ti,ab,kw OR "fixed airflow obstruction":ti,ab,kw OR "fixed airflow limitation":ti,ab,kw OR "airflow obstruction":ti,ab,kw OR "airflow limitation":ti,ab,kw OR "airway obstruction":ti,ab,kw OR "airways obstruction":ti,ab,kw OR "bronchial obstruction":ti,ab,kw OR "spirometric obstruction":ti,ab,kw OR "spirometric airflow obstruction":ti,ab,kw OR "obstructive spirometry":ti,ab,kw OR "obstructive spirometric pattern":ti,ab,kw OR "obstructive ventilatory defect":ti,ab,kw OR "obstructive ventilatory impairment":ti,ab,kw OR "post-bronchodilator obstruction":ti,ab,kw OR "post bronchodilator obstruction":ti,ab,kw OR "post-bronchodilator airflow obstruction":ti,ab,kw OR "post bronchodilator airflow obstruction":ti,ab,kw OR "FEV1/FVC":ti,ab,kw OR "FEV1 FVC":ti,ab,kw OR "FEV1/FVC ratio":ti,ab,kw OR "forced expiratory volume/forced vital capacity":ti,ab,kw OR "forced expiratory volume in one second/forced vital

capacity":ti,ab,kw OR "forced expiratory volume in 1 second/forced vital capacity":ti,ab,kw OR "chronic bronchitis":ti,ab,kw OR "chronic bronchitides":ti,ab,kw OR emphysema:ti,ab,kw OR emphysematous:ti,ab,kw OR "pulmonary emphysema":ti,ab,kw OR "lung emphysema":ti,ab,kw) AND ([mh "Diabetes Mellitus"] OR [mh "Diabetes Mellitus, Type 2"] OR [mh "Diabetes Mellitus, Type 1"] OR [mh "Diabetes Complications"] OR [mh "Prediabetic State"] OR [mh "Hyperglycemia"] OR [mh "Blood Glucose"] OR [mh "Glucose Intolerance"] OR [mh "Insulin Resistance"] OR [mh "Hemoglobin A, Glycosylated"] OR [mh "Hypoglycemic Agents"] OR "diabetes mellitus":ti,ab,kw OR diabetes:ti,ab,kw OR diabetic*:ti,ab,kw OR diabet*:ti,ab,kw OR "type 2 diabetes":ti,ab,kw OR "type-2 diabetes":ti,ab,kw OR "type II diabetes":ti,ab,kw OR "type-II diabetes":ti,ab,kw OR "type 2 diabetes mellitus":ti,ab,kw OR "type-2 diabetes mellitus":ti,ab,kw OR "type II diabetes mellitus":ti,ab,kw OR "type-II diabetes mellitus":ti,ab,kw OR T2DM:ti,ab,kw OR T2D:ti,ab,kw OR "type 2 DM":ti,ab,kw OR "type II DM":ti,ab,kw OR "non insulin dependent diabetes":ti,ab,kw OR "non-insulin dependent diabetes":ti,ab,kw OR "noninsulin dependent diabetes":ti,ab,kw OR "non insulin dependent diabetes mellitus":ti,ab,kw OR "non-insulin dependent diabetes mellitus":ti,ab,kw OR "noninsulin dependent diabetes mellitus":ti,ab,kw OR NIDDM:ti,ab,kw OR "adult onset diabetes":ti,ab,kw OR "adult-onset diabetes":ti,ab,kw OR "maturity onset diabetes":ti,ab,kw OR "maturity-onset diabetes":ti,ab,kw OR "type 1 diabetes":ti,ab,kw OR "type-1 diabetes":ti,ab,kw OR "type I diabetes":ti,ab,kw OR "type-I diabetes":ti,ab,kw OR "type 1 diabetes mellitus":ti,ab,kw OR "type-1 diabetes mellitus":ti,ab,kw OR "type I diabetes mellitus":ti,ab,kw OR "type-I diabetes mellitus":ti,ab,kw OR T1DM:ti,ab,kw OR T1D:ti,ab,kw OR "type 1 DM":ti,ab,kw OR "type I DM":ti,ab,kw OR "insulin dependent diabetes":ti,ab,kw OR "insulin-dependent diabetes":ti,ab,kw OR "insulin dependent diabetes mellitus":ti,ab,kw OR "insulin-dependent diabetes mellitus":ti,ab,kw OR IDDM:ti,ab,kw OR "new onset diabetes":ti,ab,kw OR "new-

onset diabetes":ti,ab,kw OR "incident diabetes":ti,ab,kw OR "prevalent diabetes":ti,ab,kw OR "undiagnosed diabetes":ti,ab,kw OR hyperglycemia:ti,ab,kw OR hyperglycaemia:ti,ab,kw OR hyperglycemic:ti,ab,kw OR hyperglycaemic:ti,ab,kw OR hyperglycem*:ti,ab,kw OR hyperglycaem*:ti,ab,kw OR glycemic:ti,ab,kw OR glycaemic:ti,ab,kw OR glycem*:ti,ab,kw OR glycaem*:ti,ab,kw OR "glycemic status":ti,ab,kw OR "glycaemic status":ti,ab,kw OR "glycemic control":ti,ab,kw OR "glycaemic control":ti,ab,kw OR "glucose control":ti,ab,kw OR "poor glycemic control":ti,ab,kw OR "poor glycaemic control":ti,ab,kw OR "blood glucose":ti,ab,kw OR "blood sugar":ti,ab,kw OR "plasma glucose":ti,ab,kw OR "serum glucose":ti,ab,kw OR "fasting glucose":ti,ab,kw OR "fasting plasma glucose":ti,ab,kw OR "fasting blood glucose":ti,ab,kw OR "random blood glucose":ti,ab,kw OR "postprandial glucose":ti,ab,kw OR "oral glucose tolerance":ti,ab,kw OR OGTT:ti,ab,kw OR HbA1c:ti,ab,kw OR "Hb A1c":ti,ab,kw OR A1c:ti,ab,kw OR "hemoglobin A1c":ti,ab,kw OR "haemoglobin A1c":ti,ab,kw OR "glycated hemoglobin":ti,ab,kw OR "glycated haemoglobin":ti,ab,kw OR "glycosylated hemoglobin":ti,ab,kw OR "glycosylated haemoglobin":ti,ab,kw OR "glucose intolerance":ti,ab,kw OR "impaired glucose tolerance":ti,ab,kw OR "impaired fasting glucose":ti,ab,kw OR "impaired glucose regulation":ti,ab,kw OR "impaired glucose metabolism":ti,ab,kw OR IFG:ti,ab,kw OR IGT:ti,ab,kw OR prediabetes:ti,ab,kw OR prediabetic:ti,ab,kw OR "pre diabetes":ti,ab,kw OR "pre-diabetes":ti,ab,kw OR "pre diabetic":ti,ab,kw OR "pre-diabetic":ti,ab,kw OR "prediabetic state":ti,ab,kw OR "insulin resistance":ti,ab,kw OR "insulin resistant":ti,ab,kw OR "insulin sensitivity":ti,ab,kw OR "impaired insulin sensitivity":ti,ab,kw OR "reduced insulin sensitivity":ti,ab,kw OR antidiabetic:ti,ab,kw OR anti-diabetic:ti,ab,kw OR "anti diabetic":ti,ab,kw OR "glucose lowering":ti,ab,kw OR "glucose-lowering":ti,ab,kw OR "hypoglycemic agent":ti,ab,kw OR "hypoglycemic agents":ti,ab,kw OR "hypoglycaemic agent":ti,ab,kw OR "hypoglycaemic agents":ti,ab,kw)
